# MARTX Toxin in the Zoonotic Serovar of *Vibrio vulnificus* Triggers an Early Cytokine Storm in Mice

**DOI:** 10.3389/fcimb.2017.00332

**Published:** 2017-07-20

**Authors:** Celia Murciano, Chung-Te Lee, Ana Fernández-Bravo, Tsung-Han Hsieh, Belén Fouz, Lien-I Hor, Carmen Amaro

**Affiliations:** ^1^Departamento de Microbiología y Ecología & Estructura de Recerca Interdisciplinar en Biotecnologia i Biomedicina, Universitat de València Valencia, Spain; ^2^Department of Microbiology & Immunology & College of Medicine, National Cheng Kung University Tainan, Taiwan; ^3^Institute of Basic Medical Sciences, College of Medicine, National Cheng Kung University Tainan, Taiwan

**Keywords:** *Vibrio*, *V. vulnificus*, MARTX, sepsis, infection, gene expression, qPCR array

## Abstract

*Vibrio vulnificus* biotype 2-serovar E is a zoonotic clonal complex that can cause death by sepsis in humans and fish. Unlike other biotypes, Bt2 produces a unique type of MARTX_Vv_ (Multifunctional-Autoprocessive-Repeats-in-Toxin; RtxA1_3_), which is encoded by a gene duplicated in the pVvBt2 plasmid and chromosome II. In this work, we analyzed the activity of this toxin and its role in human sepsis by performing *in vitro, ex vivo*, and *in vivo* assays. First, we demonstrated that the ACD domain, present exclusively in this toxin variant, effectively has an actin-cross-linking activity. Second, we determined that the whole toxin caused death of human endotheliocytes and monocytes by lysis and apoptosis, respectively. Finally, we tested the hypothesis that RtxA1_3_ contributes to human death caused by this zoonotic serovar by triggering an early cytokine storm in blood. To this end, we used a Bt2-SerE strain (R99) together with its *rtxA1*_*3*_ deficient mutant, and a Bt1 strain (YJ016) producing RtxA1_1_ (the most studied MARTX_Vv_) together with its *rtxA1*_*1*_ deficient mutant, as controls. Our results showed that RtxA1_3_ was essential for virulence, as R99ΔΔ*rtxA1*_*3*_ was completely avirulent in our murine model of infection, and that R99, but not strain YJ016, induced an early, strong and dysregulated immune response involving the up-regulation of a high number of genes. This dysregulated immune response was directly linked to RtxA1_3_. Based on these results and those obtained *ex vivo* (human blood), we propose a model of infection for the zoonotic serovar of *V. vulnificus*, in which RtxA1_3_ would act as a sepsis-inducing toxin.

## Introduction

*Vibrio vulnificus* is an autochthonous inhabitant of marine and estuarine waters located in tropical, subtropical, and temperate ecosystems (Oliver, 2015). Currently, the species is expanding to cooler areas such as, the Baltic Sea coast due to global warming (Baker-Austin et al., 2012). *V. vulnificus* switches between free-swimming and sessile life-forms, both establishing particular relationships with filtering organisms (mainly oysters) and fish (mainly eels), its animal reservoirs in water (Oliver, 2015). *V. vulnificus* was defined as a bacterial species in 1976 and was later split into three biotypes (Bts) on the basis of differences in phenotypic and genotypic traits as well as host range (Tison et al., 1982; Bisharat et al., 1999).

The diseases caused by *Vibrio* species are known as vibrioses. There are two main forms of human vibriosis related to the transmission route; contact (type I) or ingestion (type II). In type I, the pathogen causes severe tissue necrosis after contact of a wound with seawater or fish that can lead to debridement/amputation or even secondary septicaemia (Strom and Paranjpye, 2000; Jones and Oliver, 2009). Since people that suffer human vibriosis type I are usually swimmers, bathers and fishers, this pathogen is sometime known as the “marine flesh-eating bacterium.” In type II, the pathogen causes gastroenteritis or primary septicemia after ingestion of raw seafood. The common characteristic to both vibrioses is that they can lead to sepsis with a probability of death strongly dependent on iron levels in patient's blood (Feldhusen, 2000; Jones and Oliver, 2009; Horseman and Surani, 2011), being >50% in patients with hemochromatosis or other iron overload conditions (Horseman and Surani, 2011; Arezes et al., 2015). Fish vibriosis is a haemorrhagic septicaemia produced by contact with water or fish (carrier or diseased) without a relationship with high iron levels in blood (Amaro et al., 2015). The common feature of fish and human vibrioses is that the pathogen invades the blood causing sepsis. Regarding Bts, all three can cause human vibriosis but only Bt2 is also able to cause fish vibriosis, an ability that relies on a plasmid which encodes a resistance system to the fish innate immunity (Amaro and Biosca, 1996; Lee et al., 2008; Valiente et al., 2008b; Amaro et al., 2015). Further, among Bt2 strains, only those belonging to clonal complex Bt2-SerE (serovar) are recognized as being truly zoonotic (Amaro and Biosca, 1996; Sanjuán et al., 2011).

Among all the *V. vulnificus* virulence factors, the RtxA1 toxin seems to be the most relevant, as it is the one present in the majority of clinical strains (Satchell, 2011, 2015; Lee et al., 2013; Kim et al., 2015). In the case of human vibriosis, RtxA1 was proposed to be involved in innate immune evasion in subcutaneously infected mice (model for type I vibriosis; Lo et al., 2011) as well as in intestinal epithelium destruction plus blood invasion in intragastrically infected mice (model for type II; Kim et al., 2008; Jeong and Satchell, 2012). In the latter model, it seems that the major hemolysin produced by the three Bts of *V. vulnificus*, VvhA, could have an additive effect on RtxA1 (Jeong and Satchell, 2012). In the case of fish vibriosis, RtxA1 is involved in both defense against innate immune cells (phagocytosis experiments with primary cultures of eel's neutrophils) and animal death (virulence experiments in eels infected by intraperitoneal [i.p.] injection or immersion) (Callol et al., 2015a,b).

**Figure 1:**
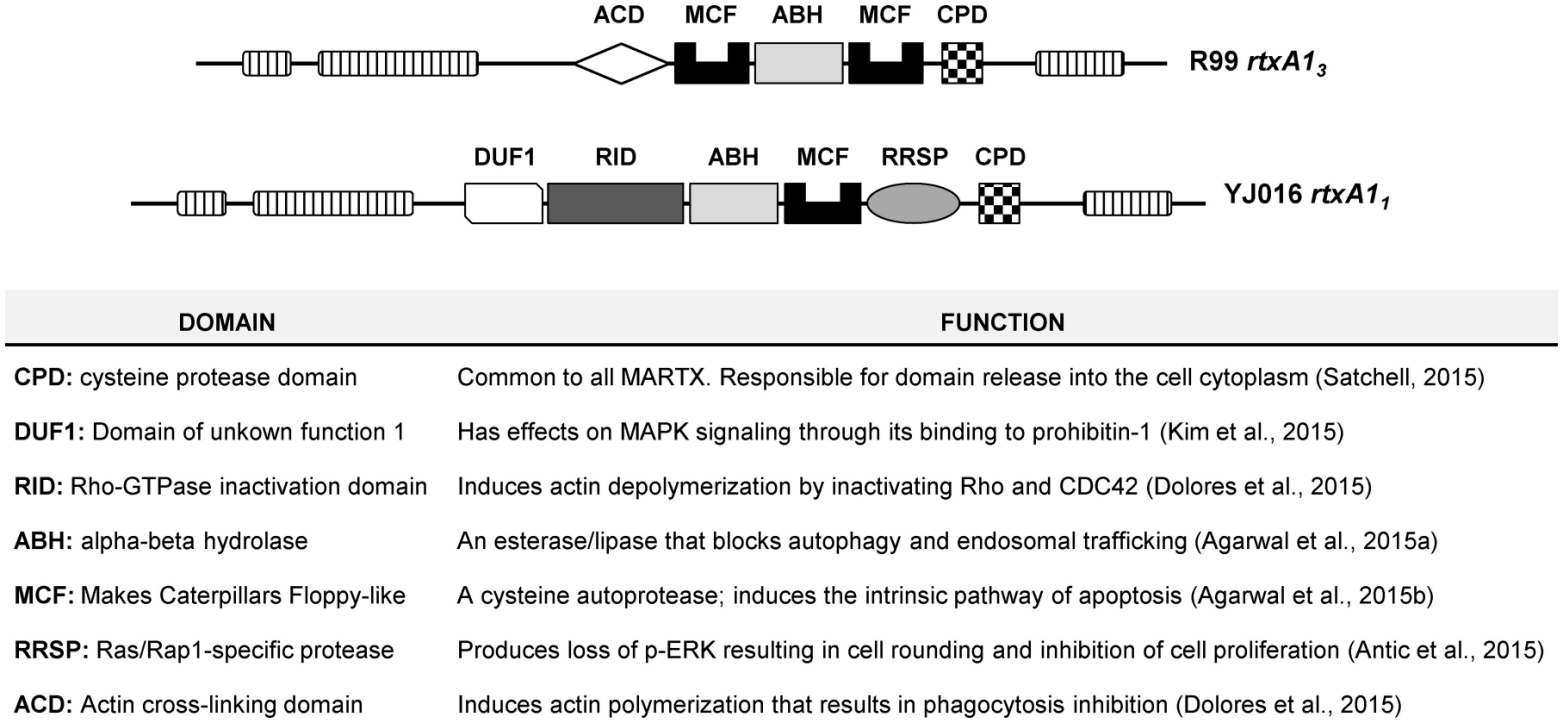
Schematic representation of type III and type I MARTX toxins, and function of each domain.

*V. vulnificus* RtxA1, or MARTX_Vv_, belongs to the subfamily of Multifunctional Autoprocessing Repeats-in-toxins (Fullner Satchell, 2007; Satchell, 2011, 2015). MARTX toxins present two common external modules containing the repeat sequences, together with a variable internal module containing a unique combination of functional domains responsible for the toxic action. The toxin is secreted after bacterium-cell contact and it associates to the eukaryotic cell membrane where the external modules form a pore for translocation of the internal module to the cytoplasm. Later, the toxin autoprocesses and the domains are liberated into the cytoplasm. Seven types of MARTX_Vv_ and nine functional domains have been described so far (Satchell, 2015). The molecular function of eight out of these nine domains has been demonstrated by Dr. Satchell's laboratory (Antic et al., 2015; Dolores et al., 2015; Kim et al., 2015; Satchell, 2015; Agarwal et al., 2015a,b) (Figure 1). All MARTX_Vv_ seem to act as cytolysins *in vitro* but differ in the mechanism of cell destruction, which probably relys on the precise domain combination (Satchell, 2015). With the exception of Bt2, there is no relationship between RtxA1 type and Bts. Figure 1 shows the two main MARTX_Vv_ types. RtxA1_1_ (or MARTX type I) is present in the most virulent Bt1 strains and its mode of action has been extensively studied (Lee et al., 2007; Liu et al., 2007; Kim et al., 2008, 2015; Kwak et al., 2011; Lo et al., 2011; Jeong and Satchell, 2012; Satchell, 2015). In contrast, RtxA1_3_ (or MARTX type III) is still uncharacterized. Interestingly, *rtxA1*_*3*_ is duplicated in the pVvBt2 plasmid and the chromosome II of Bt2 strains.

The present work is focused on RtxA1_3_ and its hypothetical role *in vivo* as a virulence factor involved in both human and fish sepsis. Our hypothesis is that once the bacterium arrives in blood, and only if it is able to resist the bactericidal/bacteriostatic action of serum, the bacterium multiplies, liberates the toxin after its interaction with blood cells, and triggers a cytokine (CK) storm that could be fatal. This hypothesis is based on the results obtained in the eel; fish i.p.-infected with mutants deficient in both copies of the toxin were colonized internally and externally by the mutant but they the eels did not develop vibriosis (Lee et al., 2013). Further, there was no statistical differences in colonization index of internal organs between wild-type and mutant strains (Lee et al., 2013), and bacterial numbers were far below those characteristic for other fish pathogenic *Vibrio* spp. (i.e., *V. anguillarum*).

To demonstrate this hypothesis, we selected the Bt2-SerE wild-type strain CECT 4999 (R99) and its derivative mutant R99ΔΔ*rtxA1*_*3*_, which lacks the two copies of *rtxA1*_*3*_, and generated three new mutants, one deficient in the ACD-domain (the only uncharacterized domain in *V. vulnificus*) (R99ΔΔ*ACD*) in *vvhA* (R99Δ*vvhA*) and in both toxins (R99ΔΔ*rtxA1*_*3*_Δ*vvhA*). We included in the study strain YJ016, a Bt1 human isolate that produces RtxA1_1_ (Bt1-RtxA1_1_; Chen et al., 2003) as representative of the most virulent lineage (Cohen et al., 2007; Sanjuán et al., 2011), as well as its derivative mutant YJ016Δ*rtxA1*_*1*_ for comparative purposes. The strains were used in the following assays: (i) *in vitro* assays to determine the molecular action of the ACD domain and the type of cell death associated to RtxA1_3_; (ii) *ex vivo* assays to compare growth in human blood/serum of Bt1-RtxA1_1_ and Bt2 strains as well as to follow the transcription of *rtxA1*_*1*_, *rtxA1*_*3*_, and *vvhA*; and (iii) *in vivo* assays in an animal model of sepsis, in which we determined the virulence degree of the wild-type strain vs. each one of the mutants and followed the transcription in blood of 84 host immune-related genes.

## Materials and methods

### Bacterial strains, plasmids, and growth conditions

The bacterial strains and their characteristics are listed in Table 1. Bacteria were routinely grown on Tryptone Soy agar supplemented with 0.5% NaCl (TSA-1) at 37°C for 24 h. Prior to mouse infection, the colony morphology on TSA-1 was checked to confirm its opaque morphology (*V. vulnificus* shifts between opaque (capsulated) and translucent (acapsulated) morphotypes, as acapsulated cells are avirulent for mice (Simpson et al., 1987). All strains were stored in LB-1 plus glycerol (17%) at −80°C.

**Table 1:** Strains used in this study.

**Designation**	**Description**	**Isolation source/References**
***V. vulnificus***		
CECT 4999^*^ (R99)	Biotype 2, serovar E (Bt2-SerE)	Diseased eel (Spain) (Lee et al., 2008)
YJ016^**^	Biotype1 (Bt1-RtxA1_1_)	Taiwan (1993) (Chen et al., 2003)
R99ΔΔ*rtxA1_*3*_*	CECT4999 *rtxA1_*3*_*-defective mutant. Lacks both plasmidic and chromosomic copies	Lee et al., 2013
R99Δ*vvhA*	CECT4999 *vvhA*-defective mutant	This study
R99ΔΔACD	CECT4999 ACD domain-defective mutant	This study
R99ΔΔ*rtxA1_*3*_ΔvvhA*	CECT4999 *rtxA1_*3*_* and *vvhA*-defective mutant	This study
YJ016Δ*rtxA1_*1*_*	YJ016 *rtxA1_*1*_*-defective mutant	Lo et al., 2011
***E. coli***		
DH5α	*SupE44 lacU169* (φ80 *lacZM15)hsdR17 recA1 endA1 gyrA96 thi-1 relA1*	Hor et al., 2000
S17-1λpir	*Thi thr leu tonA lacy supE recA*::RP4-2-Tc::Mu Km/*λpir*	Hanahan, 1983

* CECT 4999 (Spanish Type Culture Collection or CECT).

** *YJ016 [Dr. L-I Hor collection; originally isolated in the National Cheng-Kung University Hospital (Taiwan)]*.

The inoculum for *ex vivo* and *in vivo* assays was prepared as follows: one opaque colony was seeded in LB-1 (Luria-Bertani broth, 1% NaCl) and bacteria were incubated at 37°C with shaking (100 rpm) up to mid-exponential growth phase. These bacterial cells were immediately used.

### Generation of the R99ΔΔACD, R99Δ*vvhA*, and R99ΔΔ*rtxA1_*3*_*Δ*vvhA* mutants

All mutants were generated by *in vivo* allelic exchange as described previously (Shao and Hor, 2000). Briefly, to generate R99Δ*vvhA* and R99ΔΔ*rtxA1*_*3*_Δ*vvhA* mutants, DNA fragments from the *vvhA* down- and up-stream regions were amplified with the primer pairs vvhA-1/vvhA-2 and vvhA-3/vvhA-4 (Supplementary Table 1), respectively. Both fragments were sequentially cloned into pGEMT®-easy vector (Promega) and then transformed into DH5α to generate a recombinant fragment containing a 1,380 bp-deletion within *vvhA*. This recombinant fragment was removed from the pGEMT®-easy vector by enzymatic digestion at the two *Xmn*I sites, and cloned into the suicide vector pCVD442. The recombinant suicide plasmid was transformed into *E. coli* S17-1λpir and then transferred into R99 or R99Δ*rtxA1*_*3*_ by conjugation to generate the mutants by allelic exchange. Similarly, to construct the R99ΔΔ*ACD* derivative, DNA fragments from the ACD down- and up-stream regions were amplified with the primer pairs gp018/gp019 and gp020/gp021 (Supplementary Table 1), respectively, and then cloned into pUC19 between *Sac*I and *Xba*I sites. This recombinant fragment, containing a 1,392-bp deletion in ACD, was removed by *Sac*I and *Xba*I enzymatic digestion and then cloned into pCVD442. The resultant recombinant vector was transformed into *E. coli* S17-1λpir and subsequently transferred into R99 by conjugation to generate the mutant by allelic exchange. All mutants were confirmed by PCR and sequence determination.

### *In vitro* experiments

#### Cell lines, growth conditions, and *in vitro* infection

The cell lines, THP-1 (human peripheral blood monocytes, ATCC reference number TIB-202) and ECV304 (human vascular endothelial cells, ATCC reference number CRL-1998) were obtained from the Cell Culture facility of the University of Valencia (Spain). THP-1 cells were maintained as suspension cells in Roswell Park Memorial Institute Medium (RPMI-1640, Biowest Europe) supplemented with 10% FBS (fetal bovine serum, Biowest Europe) plus 1% P/S solution (penicillin-streptomycin stock, Biowest Europe), and ECV304 cells were maintained as adherent cells in DMEM (Dulbecco's Modified Eagle's Medium) supplemented with 10% FBS plus 1% P/S, in both cases at 37°C and 5% CO_2_. Three hours or 24 h prior to infection, respectively, cells were seeded in tissue culture plates (5 × 10^5^ cells mL^−1^) containing serum and antibiotic-free DMEM (serum-starvation conditions). Cells were then infected with washed bacteria from exponential cultures in serum-free DMEM at different MOIs (multiplicity of infection; bacteria:human cell).

##### Cell damage assay

Supernatants from infected ECV304 were harvested and cell damage induction was determined by lactate dehydrogenase (LDH) release using the Cytox 96 Non-Radioactive Cytotoxicity Assay kit (Promega) according to manufacturer's instructions. A recombinant bovine LDH (Sigma-Aldrich) was used to generate a standard curve and sample values were extrapolated from the curve.

#### Detection of actin polymerization by western blotting

Infected THP-1 and ECV304 cells were lysed using a modified RIPA lysis buffer (50 mM Tris-HCl, pH 7.4, 150 mM NaCl, 1 mM EDTA, 1% Triton X-100, 1% sodium deoxycholate, 0.1% SDS) containing 1X HaltTM Protease & Phosphatase Inhibitor cocktail (Sigma-Aldrich). The lysate was incubated on ice for 30 min and centrifuged for 10 min in a refrigerated microfuge. Supernatants were assayed for total protein using the BCA Protein Quantitation kit (Pierce, Thermo Scientific). Protein (15 μg) was separated on a 12% SDS-PAGE gel before transfer to an Immun-blot PVDF membrane (Roche Diagnostics). Membranes were incubated with anti-human α-actin primary (1:5000 dilution; Millipore) and secondary (1:10,000 dilution; Sigma-aldrich) antibodies and developed using Lumi-light Western Blotting substrate (Roche Diagnostics).

#### Necrosis/apoptosis assay by flow cytometry

Infected THP-1 cells were collected, washed twice with PBS and stained with Annexin-V and Propidium iodide (PI) using the FITC Annexin V Apoptosis Detection Kit I (BD Pharmingen), according to manufacturer's instructions. Apoptosis/necrosis induction was then measured by Flow cytometry using a FACSverse flow cytometer (BDbiosciences).

### *In vivo* experiments

#### Animal model of sepsis: mean lethal dose determination (LD_50_) and immune-response assays

The bacterial virulence for mice was determined in 6- to 8-week old female (mice BALB/c, Charles River, France) by i.p. injection with ten-fold serially diluted bacterial suspensions in PBS. Mortalities were recorded only if the inoculated bacterium was recovered in pure culture from kidney or liver of moribund animals. The LD_50_ was calculated as described (Reed and Muench, 1938).

To determine the immune response during sepsis, groups of 6–8-week-old female were i.p. injected with 1 × 10^6^ CFU/mouse, and mice were anesthetized at 2, 4, or 6 h post-infection. Blood was collected by cardiac puncture and samples were stored in RNA later at 4°C until RNA extraction. PBS-injected mice were used as controls. After blood extraction, mice were sacrificed and the spleen and liver were obtained to microbiologically confirm bacterial invasion. To this end, spleens and livers were mechanically disaggregated in 1 mL of PBS. The homogenate was inoculated completely into LB-1 media and was incubated overnight at 37°C with shaking. In parallel, a loop of the homogenate was directly streaked onto TSA plates. After incubation, 0.1 mL of the LB-1 culture was spread onto TSA-1 plates. All seeded TSA-1 plates were incubated at 37°C for 24 h. Plates were examined to confirm purity of the culture and randomly selected colonies were purified and serologically identified by agglutination with previously obtained specific anti-YJ016 or anti-R99 rabbit polyclonal antibodies.

#### Murine RNA processing for gene expression analysis

Total RNA was isolated using the Mouse RiboPure™ -Blood RNA Isolation Kit (Invitrogen, Thermo Fisher Scientific) and treated for 45 min at 37°C with Turbo DNase™ (Ambion®, Life Technologies). The concentration and purity of each RNA was checked in a Nanodrop 2000 Spectrophotometer (Thermo Scientific). To check the absence of genomic DNA, real-time qPCR was performed on the RNA samples by using Power SYBR® green PCR Mastermix (Applied Biosystems®, Life Technologies) and *B2m* and *Gusb* primers. The Real-time qPCR was performed on a StepOnePlus™ Real-Time PCR System (Applied Biosystems), with the primers were taken from Primerbank. RNA from each mice group was then pooled after discarding samples that presented low quality/gDNA contamination. An RNA pool from a minimum of 3 animals was used to obtain the cDNA. The cDNA was transcribed from total RNA using All-in-One™ First-Strand cDNA Synthesis Kit (GeneCopoeia™).

#### Murine gene expression analysis by qPCR array

To assess the global immune response activated by *V. vulnificus* during an *in vivo* infection, we designed a specific qPCR array for profiling the expression of 84 immune-related genes (Supplementary Table 2) (ExProfile Custom Gene qPCR Array, GeneCopoeia™). The PCR array included probes for genes involved in the general immune response against bacteria as well as genes involved in the specific immune response in eels against *V. vulnificus* (Callol et al., 2015b & Unpublished results). The selected genes included: 12 genes for CCs, 18 genes for CKs, 8 genes related with the IFN response, 5 genes for transcription factors, 15 genes for pathogen recognition receptors (PRRs) and other cell receptors, 8 genes for signaling proteins, 5 genes related with the inflammasome response and 13 additional genes that included genes for complement proteins, apoptosis related genes, iron starvation, etc. (Supplementary Table 2). Real-time PCR was performed on cDNA using All-in-One™ qPCR Mix (GeneCopoeia™) and the customized Gene qPCR Array (GeneCopoeia™), on a StepOnePlus™ Real-Time PCR System. The threshold cycle (CT) values were determined to establish the relative RNA levels of the tested genes, and the fold-change in gene expression of *V. vulnificus* infected mice compared with uninfected control mice was calculated using the GeneCopoeia analysis tool for custom arrays. Six different housekeeping genes included in the array (Supplementary Table 2) were used in the analysis. Each treatment (strain and/or infection time) was performed twice, on separate days, and assessed in two different arrays independently, before being analyzed together as replicates with the GeneCopoeia analysis tool.

#### Expression of *rtxA1*_3_ *in vivo*

The expression of *rtxA1*_3_ and *vvha* genes was also determined in the blood samples from *V. vulnificus* R99 infected mice. The real-time qPCR was performed on the same cDNA samples used for the customized gene qPCR array. Real-time PCR was performed on cDNA using Power SYBR® green PCR Mastermix on a StepOnePlus™ Real-Time PCR System. The CT values were determined with StepOne Software v2.0 to establish the relative RNA levels of the tested genes, using recA gene as housekeeping gene. In Bt2-SerE infected mice, the value of expression of each gene in mice infected for 2 h was set equal to 1, and then the fold increase in gene expression was calculated in mice infected for 4 and 6 h. In Bt1-RtxA1_1_ infected mice, the value of expression of rtxA1_1_ in mice infected with the YJ016ΔrtxA1_1_ mice was set equal to 1, and then the fold increase in gene expression was calculated in mice infected with the YJ016 strain. The bacterial primers used are listed in Supplementary Table 1.

#### Analysis of data

We created Venn diagrams using the software available at http://bioinformatics.psb.ugent.be/webtools/Venn/ (Bioinformatics and Evolutionary Genomics group at Ghent University). Heat maps were created using the free software MultiExperiment Viewer (MeV), available at http://mev.tm4.org/. For PCA, we created a correlation matrix using Past v3.13 program (Hammer et al., 2001). Then a 3D graph was generated using the excel macro “Excel 3D Scatter Plot' v2.1”, available at http://www.doka.ch/Excel3Dscatterplot.htm.

### *Ex vivo* experiments

#### Model for hemochromatosis (hm)

The *ex vivo* model for hm consisted of supplementing commercial human serum (Sigma-Aldrich, Spain) with FeCl_3_ at a final concentration of 10 μM (iron content within the range of those typical of hemochromatosis) (Bacon et al., 2011) (hm-serum). Then, hm-blood was prepared by resuspending human blood cells in hm-serum. To this end, whole human blood was obtained from volunteers recruited from the University of Valencia. Blood was collected by venipuncture using lithium heparin tubes (BD Vacutainer®, BD Biosciences). Blood cells were separated by centrifugation, erythrocytes were lysed with ACK buffer (0.15 M NH_4_Cl, 1 0mM KHCO_3_, 1 mM EDTA), and the number of blood cells was determined by microscopic counting. Finally, 2.5 × 10^6^ blood cells were resuspended in 150 μl of hm-serum. Hm-blood and hm-serum were immediately used.

#### Hm-serum resistance and *rtxA1*_3_ and *vvha* expression in hm-blood

Growth in normal and hm-serum was performed by inoculating R99 or YJ016 cells at a MOI (multiplicity of infection; bacteria: human cell) of 2, and taking samples for bacterial counts on TSA-1 plates at 0, 2, 4, and 6 h. *rtxA1*_*1*_ and *rtxA1*_*3*_ expression was followed in normal and hm-blood by taking samples at the same time intervals. Cells were pelleted and the RNA was isolated. Different RNA isolation kits were tested beforehand, and the GenElute™ Mammalian Total RNA Miniprep Kit (Sigma-Aldrich) was found to produce higher yields and better RNA purity. RNA was treated with Turbo DNase™, and cDNA was transcribed from total RNA using random hexonucleotide primers (Takara Bio Europe), and Maxima™ H Minus Reverse Transcriptase (Thermo Scientific). Real-time PCR was performed and analyzed as described above, and the bacterial primers used are listed in Supplementary Table 1.

### Ethics statement

Whole human blood was obtained from healthy volunteers recruited among the staff of the Microbiology & Ecology Department from the University of Valencia. Donors gave their written informed consent to participate in the study, in accordance with the Declaration of Helsinki. They completed a health questionnaire, and doctor's consent for their inclusion in the study was obtained. Only healthy donors were considered; volunteers with anti-inflammatory or immunosuppressive prescribed medication, diabetes mellitus, chronic inflammatory disease, or symptoms of recent infection were excluded. Volunteers were recruited specifically for the purpose of this experiment, and blood was collected at the Burjassot campus medical service of the University of Valencia. Assays were approved by the Institutional Committee on Human Research (project license H1487946643442).

All assays involving mice were approved by the Institutional Animal Care and Use Committee and the local authority (Conselleria de Agricultura, Medio Ambiente, Cambio Climático y Desarrollo Rural. Generalitat Valenciana), following European Directive 2010/63/EU and the Spanish law “Real Decreto” 53/2013. Murine infections were performed under the project licenses 2014/VSC/PEA/00195 and 2016/VSC/PEA/00069, and were carried out in the Research Animals core facility of the University of Valencia.

The experiments performed in this study are part of the working plan that was approved and supported by the Ministry of Economy Industry and Innovation (MINECO) of Spain: Grant AGL 2014-58933-P.

## Results

### RtxA1_3_ but not VvhA is involved in virulence for mice

The LD_50_ of R99 (Bt2-SerE) and YJ016 (Bt1-RtxA1_1_) strains was identical (1 × 10^6^ CFU/mouse or 5 × 10^4^ CFU/gr). The deletion of *vvhA* in R99 strain did not change LD_50_ value, while the deletion of both copies of *rtxA1*_*3*_, or both copies plus *vvhA*, caused an increase in more than 2 logarithmic units of the LD_50_(>10^8^ CFU/mouse). Previously, our group had shown that the double revertant strain (with both copies of *rtxA1*_*3*_ re-introduced) had the same virulence level in C3H/HeN mice as the wild-type strain, confirming that the reduction in virulence of the RtxA1_3_ mutant was not caused by an unexpected mutation that had occurred elsewhere (Lee et al., 2013). These results suggest that RtxA1_3_ but not VvhA is involved in mouse death caused by Bt2-SerE strains.

### RtxA1_3_ is a toxin with actin-cross-linking activity, responsible for early death of human endotheliocytes and monocytes *in vitro*

**Figure 2:**
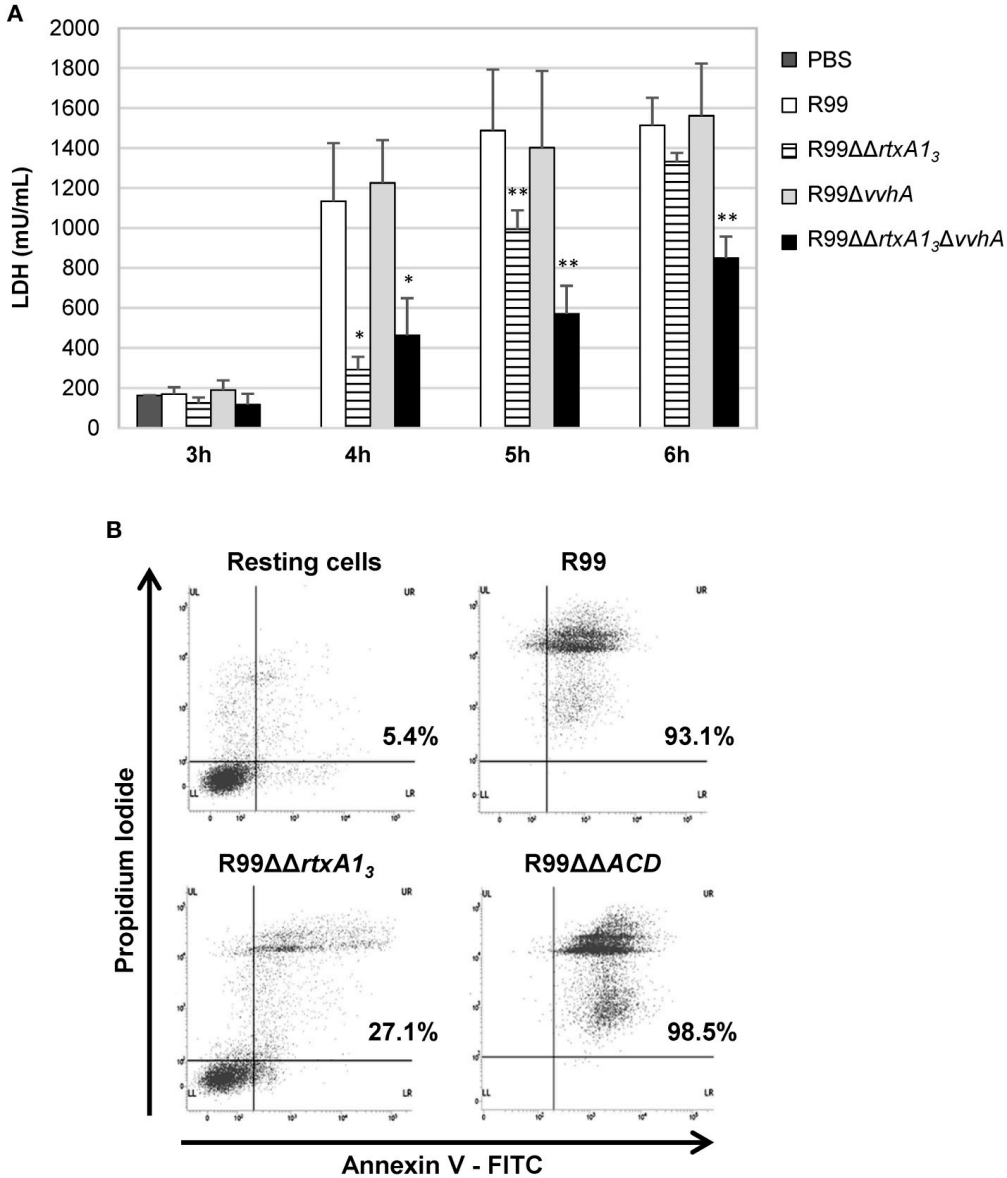
*V. vulnificus rtxA1*_3_ gene is involved in cell damage induction and apoptosis in human monocytes. **(A)** Live cells of *V. vulnificus* R99, R99ΔΔ*rtxA1*_*3*_ mutant, R99Δ*vvha* mutant, and the double R99ΔΔ*rtxA1*_*3*_Δ*vvhA* mutant, were added at a MOI of 2 to ECV304 cells. Cells were incubated with the bacteria for the appropriate time intervals and then cell culture supernatants were collected and assessed for lactate dehydrogenase (LDH) levels. Data represent mean values ± standard deviation from three independent experiments. The significance of the differences was determined using the Student's two-tailed *t*-test; ^*^p < 0.05 and ^**^p < 0.01 compared with R99 infected cells. **(B)** THP-1 cells were infected for 3.5 h with *V. vulnificus* R99, R99ΔΔ*rtxA1*_*3*_, and R99ΔΔ*ACD* strains, at a MOI of 10. The induction of apoptosis/necrosis was then measured by Flow Cytometry using the FITC Annexin V Apoptosis Detection Kit I. Results show histograms from one representative experiment of two.

RtxA1_3_ from Bt2-SerE has been reported to be involved in early cell death (Lee et al., 2013). To eliminate the possibility that VvhA could interfere with RtxA1_3_ in Bt2-SerE, we infected human vascular endotheliocytes (ECV304 cell line) and monocytes (THP-1 cell line) with R99 strain and its derivative mutants (R99ΔΔ*rtxA1*_*3*_, R99Δ*vvhA*, R99ΔΔ*rtxA1*_*3*_Δ*vvhA*) and incubated the infected cells for 6 h. The R99 strain started to lyse cells before 4 h post-infection (hpi) with a maximum at 5 h that was maintained up to 6 hpi, without significant differences with R99Δ*vvhA* strain at any sampling point. In contrast, there were significant differences in cell lysis between R99 and R99ΔΔ*rtxA1*_*3*_ strains at 4 and 5 hpi, and between R99 and R99ΔΔ*rtxA1*_*3*_Δ*vvhA* strains at 4, 5, and 6 hpi (Figure 2A). Similar results were obtained by infecting human monocytes (data not shown). Taken together, our results suggest that both toxins do not overlap at short term (before 6 hpi), with RtxA1_3_ the main factor responsible for early cell lysis caused by Bt2-SerE *in vitro*. Finally, a light lytic effect was also found at 6 hpi when the mutant in both toxins was tested, indicating that other toxins apart from RtxA1_3_ and VvhA could also produce cell death *in vitro*.

To find out if RtxA1_3_ could cause cell death by apoptosis, we analyzed it by flow cytometry (Figure 2B and data not shown). After 3.5 h of *in vitro* infection of human monocytes, the percentage of cells in late apoptosis (stained with both annexin V and PI) was close to 100% in the wild-type strain, whereas only 27% of the cells were in this stage after being infected with the *rtxA1*_*3*_ mutant (Figure 2B). No evidence of cell death by apoptosis was observed in endothelial cells (data not shown). This result suggests that RtxA1_3_ can induce early apoptosis in human monocytes but not in endotheliocytes cultured *in vitro*.

**Figure 3:**
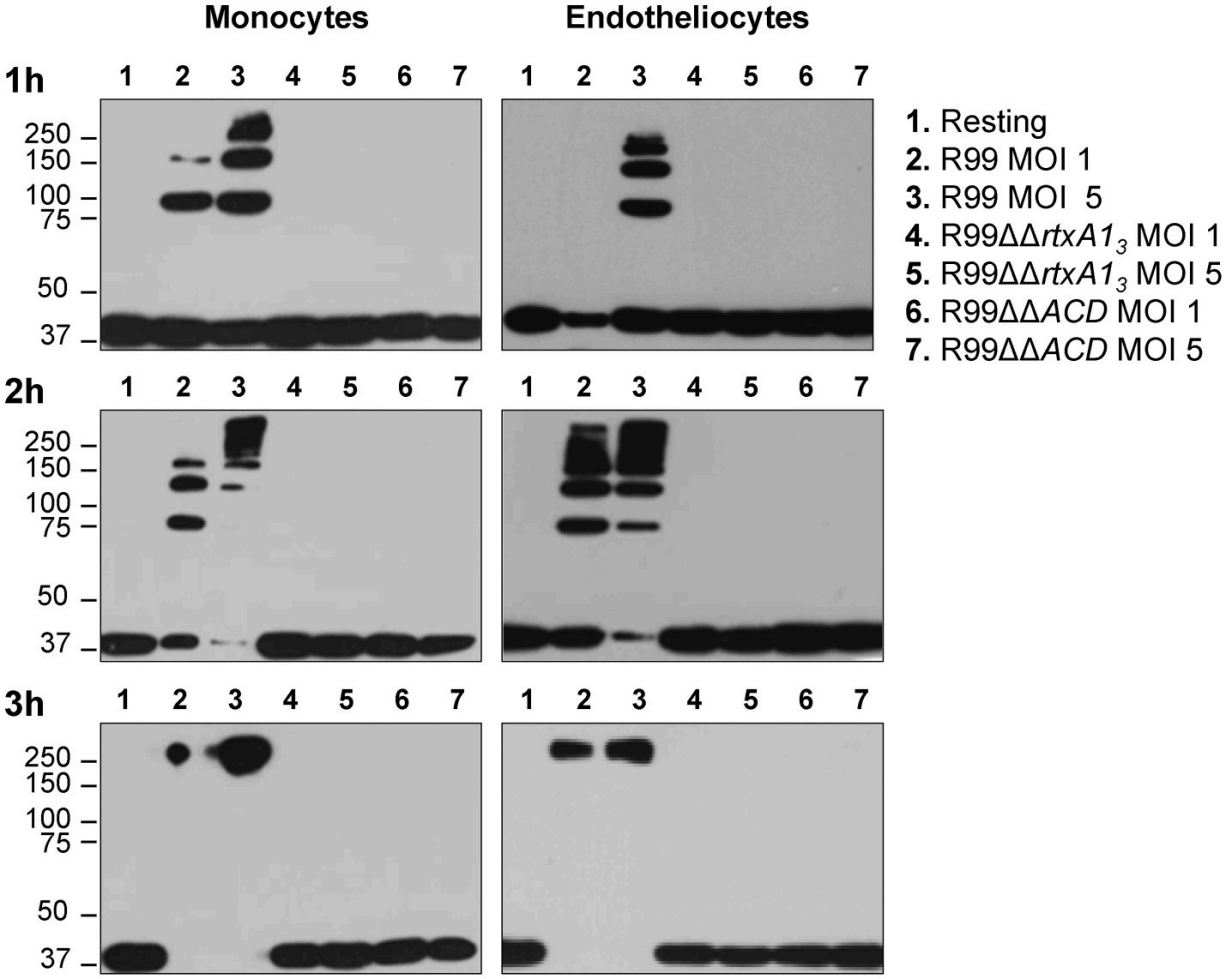
Role of the ACD domain in actin polymerization. *V. vulnificus* R99, R99ΔΔ*rtxA1*_*3*_, and R99ΔΔ*ACD* cells were added at MOIs of 1 and 5 to THP-1 suspension cells and ECV304 adherent cells for 1, 2, and 3 h. Cell lysates were separated by SDS-PAGE and western blotted to detect polymerization of α-actin. Data are representative of two independent experiments.

RtxA1_3_ contains an ACD domain that is predicted to have actin cross-linking activity due to its high similarity with the ACD domain of MARTX of *V. cholerae* (Sheahan et al., 2004; Kwak et al., 2011; Roig et al., 2011). Thus, we next sought to determine if the ACD domain and, in consequence, RtxA1_3_, could also have this activity by infecting human monocytes and endotheliocytes with R99, R99ΔΔ*rtxA1*_*3*_, and R99ΔΔ*ACD* strains and detecting actin cross-linking by western blotting (Figure 3). As early as 1 hpi, R99 strain induced the formation of actin oligomers that was dependent on the infection dose, and progressed with the infection time (Figure 3). Thus, at 3 hpi, any native actin (42 Kda) was left in the samples had all been converted to an oligomer of ~250 KDa (Figure 3). As expected neither R99ΔΔ*rtxA1*_*3*_ nor R99ΔΔ*ACD* strains induced actin polymerization, at any sampling point. All these results proved that ACD domain of *V. vulnificus* and, in consequence, RtxA1_3_, has actin-cross linking activity.

We also tested if this domain was essential to induce apoptosis in human monocytes (Figure 2B). The mutant in the ACD domain produced the same degree of apoptosis as the wild-type strain, which suggests that this domain is not involved in apoptosis of human monocytes *in vitro*.

### *V. vulnificus* Bt2-SerE r99, but not Bt1-RtxA1_1_ YJ016, induces an early and strong immune response in mice

In order to test if *V. vulnificus* Bt2-SerE induces an early and strong immune response in mice that could be compatible with a CK storm (CKS), we analyzed the transcription of 84 murine genes in blood from infected and non-infected mice by using a sepsis model of infection. We also inoculated mice with YJ016 for comparative purposes.

Prior to the expression analysis, we checked that the inoculated strain had colonized the infected animals internally by isolating it from spleen, liver, and blood. Both strains were recovered in pure culture from all the internal organs of the infected mice but only after enrichment, which prevented us from calculating an organ colonization index. No bacteria were recovered from internal organs of non-infected mice.

**Figure 4:**
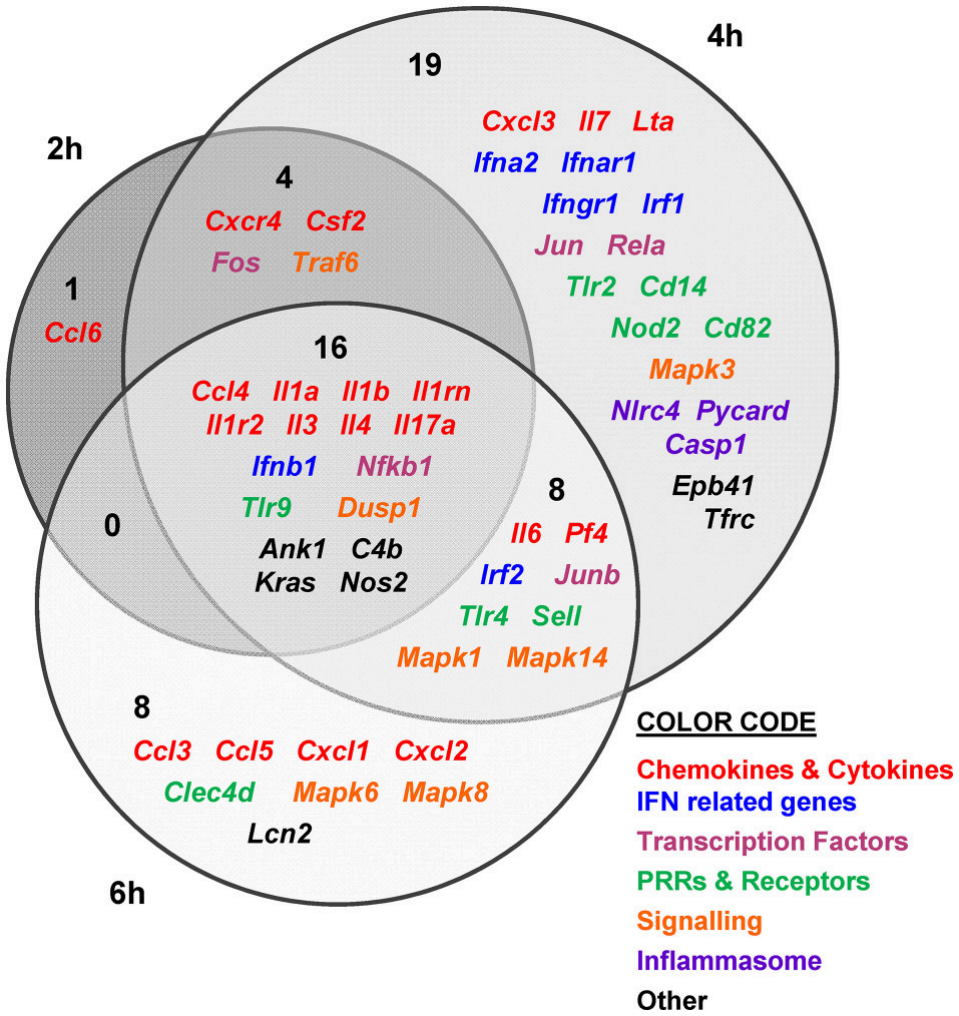
*V. vulnificus* Bt2-SerE R99 strain induces the early up-regulation of a large number of immune-related genes. Venn diagram depicting the overlap of the 56 immune-related genes that are differently up-regulated between R99 strain infected-mice for 2, 4, or 6 h, when compared with uninfected mice. Only genes up-regulated more than 1.3 were considered.

**Figure 5:**
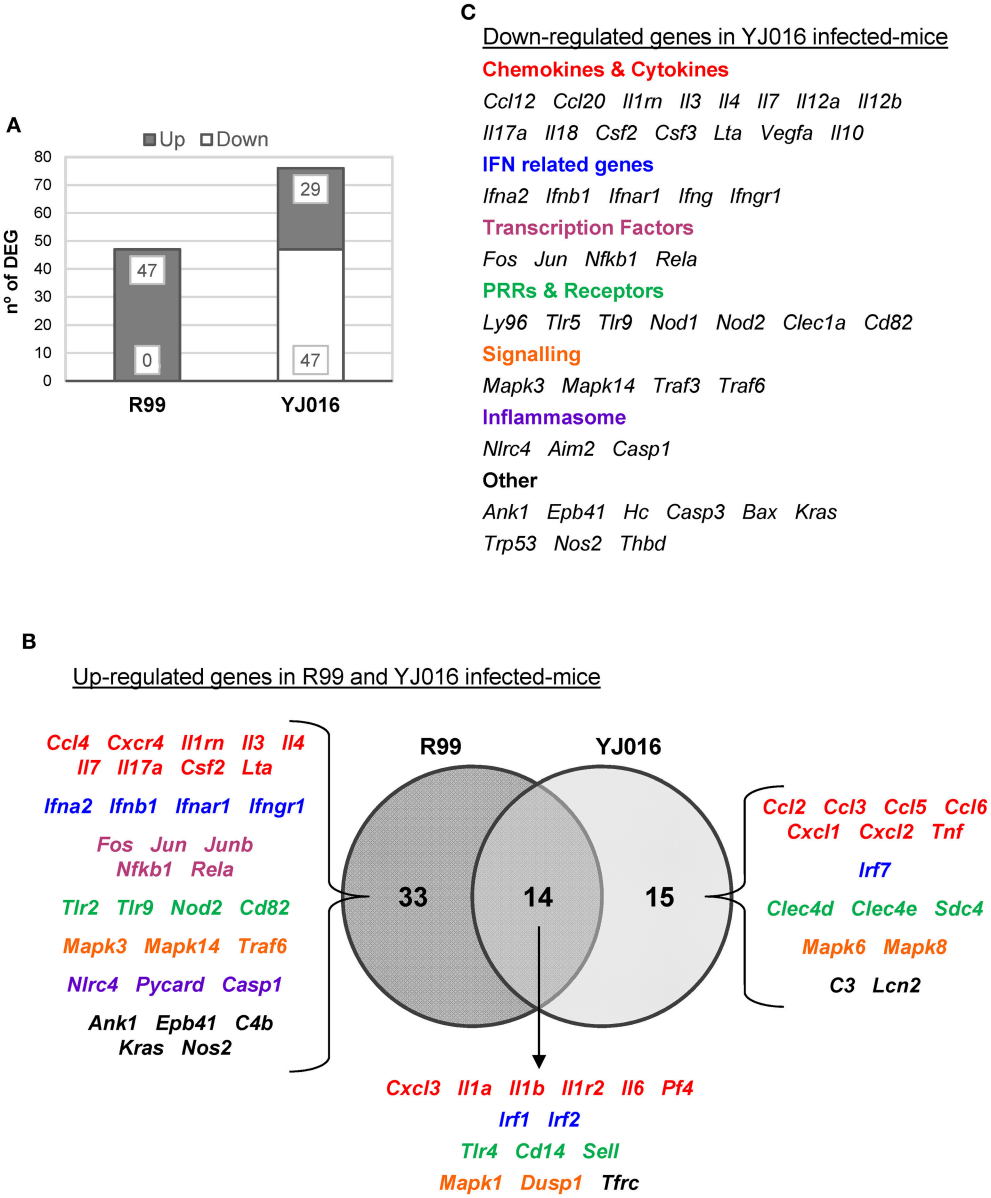
Bt1-RtxA1_1_ and Bt2-SerE elicit a different immune response. **(A)** Number of genes up- and down-regulated in mice infected with the Bt2-SerE R99 strain and the Bt1-RtxA1_1_YJ016 strain. **(B)** Venn diagram depicting the overlap of the up-regulated genes in response to R99 and YJ016 strain when compared with uninfected mice. **(C)** List of genes down-regulated after the infection with the Bt1-RtxA1_1_ YJ016 strain. In all cases, only genes up-regulated more than 1.3 or down-regulated more than 0.5 were considered.

Then, we determined murine DEGs (differentially expressed genes) in response to each of the strains by comparing data between infected and non-infected mice. Results are summarized in Figures 4, 5.

The immune response against R99 strain was very fast and strong, and remained over time: it included 56 out of the 84 selected genes (67%), all of them up-regulated (Table 2), from which 21 (out of 56 or 37.5%) were already up-regulated at 2 hpi, and 16 of them (out of 21, or 76.2%) continued to be up-regulated at 6 h (Figure 4). The core DEGs (genes up-regulated at all time points) contained genes for: (i) two interleukins with a strong pro-inflammatory and immune-regulatory activity (*Il1a, Il1b*) plus their receptor (*Il1r2*) and its agonist (*Il1rn*); (ii) four CKs and chemokines (CCs) (*Ccl4, Il3, Il4*, and *Il17a*); (iii) one type I interferon (IFN) (*Ifnb1*); (iii) Nfkb1, a subunit of the transcription factor complex NF-κB; (iv) *Tlr9*, an intracellular PRR that recognizes CpG motifs of microbial DNA; (v) *Dusp1* (MKP-1), the dual specificity protein phosphatase 1, a key phosphatase involved in a negative feedback loop regulating the MAPK pathway; (vi) *Ank1*, a protein with roles in cell motility, proliferation or cell contact; (vii) *C4b*, the complement component 4; (viii) *Kras*, a GTPase; and, ix) *Nos2*, the inducible nitric oxide synthase.

**Table 2:** Fold change value of genes when compared with uninfected mice.

	**R99**			**YJ016 (4 h)**	**R99 Δ*rtxA1_*3*_* (4 h)**	**YJ016 Δ*rtxA1_*1*_* (4 h)**
	**2 h**	**4 h**	**6 h**			
**CHEMOKINES**						
*Ccl2*	0.900	0.990	0.712	**2.613**	0.616	**0.130**
*Ccl3*	0.900	0.990	**1.425**	**21.010**	**1.748**	**0.368**
*Ccl4*	**10.157**	**3.940**	**7.799**	1.249	1.181	**0.497**
*Ccl5*	0.601	0.986	**3.827**	**4.945**	**9.320**	**3.907**
*Ccl6*	**5.078**	0.702	0.972	**2.512**	**1.666**	**1.403**
*Ccl12*	0.900	0.990	0.688	**0.115**	**0.148**	**0.062**
*Ccl20*	0.900	1.008	0.688	**0.041**	**0.151**	**0.062**
*Cxcl1*	0.882	0.992	**2.818**	**41.006**	**3.425**	0.509
*Cxcl2*	0.943	1.041	**2.864**	**41.896**	**3.479**	0.518
*Cxcl3*	0.884	**1.404**	0.676	**41.053**	**2.404**	0.507
*Pf4*	0.882	**2.038**	**22.307**	**231.676**	**433.480**	**367.456**
*Cxcr4*	**5.074**	**2.798**	0.960	1.252	**1.665**	0.991
**CYTOKINES**						
*Il1a*	**3.612**	**2.802**	**2.791**	**20.162**	**3.362**	**0.354**
*Il1b*	**1.804**	**1.983**	**2.765**	**20.033**	**26.816**	**7.953**
*Il1rn*	**1.817**	**1.993**	**1.394**	**0.080**	**0.301**	**0.125**
*Il1r2*	**1.840**	**2.035**	**1.438**	**7.244**	**6.848**	**2.870**
*Il3*	**2.540**	**1.985**	**1.952**	**0.056**	**0.295**	**0.178**
*Il4*	**3.620**	**1.988**	**1.371**	**0.039**	**0.297**	**0.088**
*Il6*	1.282	**1.992**	**7.729**	**2.514**	**0.420**	**0.125**
*Il7*	1.276	**1.394**	0.982	**0.056**	**0.297**	**0.176**
*Il12a*	0.900	0.990	0.688	**0.057**	**0.148**	**0.180**
*Il12b*	0.900	0.990	0.688	**0.057**	**0.148**	**0.064**
*Il17a*	**3.602**	**4.000**	**1.397**	**0.111**	**0.149**	**0.177**
*Il18*	0.900	1.010	0.688	**0.163**	**0.308**	**0.259**
*Csf2*	**1.841**	**1.437**	0.705	**0.160**	**0.303**	**0.128**
*Csf3*	0.900	1.013	0.688	**0.325**	**0.151**	**0.064**
*Tnf*	0.939	1.011	0.721	**2.610**	1.232	0.520
*Lta*	0.900	**1.426**	0.688	**0.058**	**0.152**	**0.090**
*Vegfa*	0.636	0.700	**0.061**	**0.020**	**0.075**	**0.044**
*Il10*	**0.446**	0.989	0.687	**0.039**	**0.209**	**0.124**
**IFN RELATED GENES**						
*Ifna2*	0.939	**1.441**	0.688	**0.039**	**0.221**	**0.090**
*Ifnb1*	**1.822**	**1.396**	**2.788**	**0.040**	**0.429**	**0.127**
*Ifnar1*	0.637	**1.392**	0.976	**0.111**	**0.417**	**0.176**
*Ifng*	0.900	0.990	0.688	**0.116**	**0.151**	**0.063**
*Ifngr1*	1.281	**1.975**	**0.487**	**0.445**	0.595	**0.354**
*Irf1*	0.919	**1.410**	0.675	**5.167**	**2.426**	**1.443**
*Irf2*	0.916	**1.397**	**1.395**	**2.559**	**3.403**	**2.026**
*Irf7*	0.900	0.990	0.688	**1.864**	1.241	0.520
**TRANSCRIPTION FACTORS**						
*Fos*	**2.573**	**1.984**	0.994	**0.079**	**0.298**	**0.125**
*Jun*	0.899	**1.985**	0.689	**0.111**	**0.147**	**0.124**
*Junb*	0.893	**1.403**	**2.769**	0.630	0.833	**0.351**
*Nfkb1*	**7.268**	**3.969**	**1.377**	**0.080**	**0.424**	**0.177**
*Rela*	**0.448**	**1.397**	0.682	**0.111**	**0.295**	**0.175**
**PRRS AND RECEPTORS**						
*Tlr2*	**0.428**	**1.982**	0.685	0.626	**0.416**	**0.248**
*Tlr4*	0.944	**2.083**	**1.435**	**1.314**	**1.748**	0.735
*Cd14*	0.900	**1.443**	0.688	**5.219**	**1.737**	**0.365**
*Ly96*	0.900	0.987	**0.171**	**0.028**	**0.104**	**0.031**
*Tlr5*	0.900	1.008	0.688	**0.041**	**0.215**	**0.064**
*Tlr9*	**3.628**	**1.989**	**1.369**	**0.111**	0.596	**0.249**
*Tlr13*	0.900	0.990	0.688	0.926	**2.464**	1.036
*Nod1*	0.900	0.990	0.688	**0.164**	**0.153**	**0.184**
*Nod2*	0.945	**2.079**	0.729	**0.234**	**0.310**	**0.090**
*Clec1a*	0.900	1.008	0.688	**0.039**	**0.148**	**0.063**
*Clec4d*	0.937	1.009	**1.434**	**14.828**	**27.825**	**11.677**
*Clec4e*	0.900	1.015	0.710	**10.415**	**13.945**	**4.134**
*Sdc4*	0.652	0.990	**0.496**	**5.073**	0.852	0.506
*Sell*	0.939	**1.463**	**1.426**	**5.213**	**6.944**	**4.130**
*Cd82*	0.893	**1.393**	0.688	**0.224**	**0.297**	**0.177**
**SIGNALING**						
*Mapk1*	0.901	**1.987**	**11.020**	**3.553**	**6.653**	**3.968**
*Mapk3*	1.277	**1.393**	0.974	**0.441**	1.179	**0.495**
*Mapk6*	0.900	0.990	**1.431**	**1.851**	**3.484**	**2.069**
*Mapk8*	0.900	1.016	**2.865**	**1.860**	**2.462**	**2.084**
*Mapk14*	1.275	**1.986**	**1.968**	**0.159**	**0.422**	**0.175**
*Dusp1*	**3.669**	**1.982**	**1.406**	**10.231**	**4.817**	**2.025**
*Traf3*	0.900	1.015	0.688	**0.233**	**0.311**	**0.184**
*Traf6*	**7.377**	**1.440**	0.671	**0.040**	**0.430**	**0.176**
**INFLAMMASOME**						
*Nlrp3*	0.900	0.990	0.688	0.925	0.616	**0.183**
*Nlrc4*	0.917	**1.428**	0.701	**0.080**	**0.151**	**0.124**
*Aim2*	0.900	1.009	0.688	**0.327**	**0.436**	0.733
*Pycard*	0.635	**1.369**	**0.486**	0.883	**3.337**	**1.981**
*Casp1*	0.619	**1.398**	**0.494**	**0.449**	1.200	0.506
**OTHER**						
*Ank1*	**5.078**	**3.958**	**1.936**	**0.111**	0.588	**0.353**
*Epb41*	1.259	**1.966**	**0.474**	**0.317**	0.595	**0.176**
*C3*	0.900	0.990	0.733	**5.255**	**9.860**	1.035
*C4b*	**2.549**	**1.397**	**1.943**	0.881	1.180	**0.496**
*Hc*	0.880	1.016	0.673	**0.057**	**0.211**	**0.088**
*Casp3*	0.888	0.991	0.686	**0.440**	1.180	**0.494**
*Bax*	0.892	0.991	0.689	**0.080**	**0.209**	**0.088**
*Kras*	**1.809**	**1.978**	**1.387**	**0.222**	0.593	**0.250**
*Trp53*	0.637	0.967	**0.465**	**0.440**	0.588	**0.496**
*Lcn2*	0.900	1.015	**91.646**	**83.699**	**111.587**	**46.747**
*Tfrc*	0.879	**1.398**	0.700	**2.559**	**4.823**	**1.435**
*Nos2*	**5.117**	**3.950**	**1.958**	**0.055**	**0.419**	**0.176**
*Thbd*	0.895	0.988	0.687	**0.019**	**0.070**	**0.124**

*Genes up-regulated more than 1.3 or down-regulated more than 0.5 are marked in bold*.

Among the DEGs that were up-regulated exclusively at 4 h were genes for: (i) another type I IFN (*Ifna2*) and its receptor (*Ifnar1*), the receptor for IFN-γ (*Ifngr1*) and an IFN regulatory factor (*Irf1*); (ii) one CC involved in the recruitment and activation of granulocytes (*Cxcl3*) and two pro-inflammatory CKs (*Il7, Lta*); (iii) three proteins of the inflammasome complex *(Nlrc4, Pycard*, and *Casp1*); (iv) two transcription factors (*Jun* and *Rela*); (v) four PRR and receptors (*Tlr2, Cd14, Nod2, Cd82)* and (iv) other genes such as, *Tfrc*, the transferrin receptor (Figure 4).

Finally, at 6 hpi there were still 32 genes up-regulated (out of 56, or 57%), 8 of them exclusively at this time. These included the genes for: (i) the CCs CCL3 (MIP-1α) and CCL5 (RANTES), involved in the recruitment and activation of different immune cells; (ii) the CXC CCs, CXCL1, and CXCL2, which are chemo-attractant for neutrophils, granulocytes and hematopoietic stem cell, respectively; (iii) the C-type lectin CLEC4D; and (iv) LCN2, a protein that limits bacterial growth by sequestrating iron.

The maximum number of up-regulated genes was reached at 4 hpi, at which time a total of 47 genes were activated (Figure 4). For that reason, we selected this time point for the immune response analysis against the selected Bt1- RtxA1_1_ strain.

Strain YJ016 induced a completely different immune response that involved a higher number of DEGs (76 vs. 47) most of them down-regulated (Figure 5). Thus, only 29 out of 76 genes were up-regulated, 14 in common with strain R99 (Figure 5B). Again, the genes for IL-1α, IL-1β, and the IL-1 receptor, belonging to the IL-1 family, were up-regulated, stressing its importance as an immune-mediator system against any *V. vulnificus* strain. Also, the genes for the CK IL-6 and for the CCs CXCL3 and PF4 (or CCXL4) were up-regulated. PF4 showed the highest value of fold-change, 232, a value that was significantly higher than the one induced by strain R99 (2- and 22-fold-change at 4 and 6 h, respectively) (Table 2). PF4, a CXC CC produced by activated platelets during its aggregation, has a large number of biological effects such as, activation of different immune cells (NK cells, T cells, monocytes, and granulocytes), differentiation and chemotaxis, inhibition of endothelial cell migration, a dual pro- and anti-coagulant effect mediated by platelet-aggregation potentiation (Sandset, 2012) and Protein C activation (Slungaard and Key, 1994), respectively. Other genes induced by both strains were the IFN regulatory factors *Irf1* and *Irf2*, the lipopolysaccharide receptors *Tlr4* and *Cd14*, and *Dusp1*.

Among the genes uniquely induced by YJ016 strain were *Ccl2* and *Tnf*α (Figure 5B). Remarkably, the pattern of induction of PRRs was very different between both strains; YJ016 induced strongly the expression of the C-type lectin genes *Clec4d* and *Clec4e*, and Syndecan-4 (*Snd4*) whereas R99 induced *Tlr2* and genes for intracellular receptors such as, NOD2 (located in the cytoplasm and recognizing muramyl dipeptide from gram-positive and negative bacteria; Kawai and Akira, 2009) and TLR9. Indeed, the genes for these two intracellular receptors were down-regulated by YJ016 strain (Figure 5C). There were also other genes up-regulated by YJ016 strain at 4 hpi that were not induced by the R99 strain at this time point, but that were induced by the R99 strain at 2 or 6 hpi, such as, *Ccl3, Ccl5, Ccl6, Cxcl1, Cxcl2, Mapk6, Mapk8*, and *Lcn2*, which was strongly up-regulated by both strains (Figures 4, 5, and Table 2).

Taking a closer look at the genes down-regulated by strain YJ016 (Figure 5C), we found genes that were up-regulated by the R99 strain, such as, the transcription factors genes *Fos, Jun, Nfkb1*, and *RelA*, a number of CCs and CKs genes (*Il1rn, Il3, Il4, IL7, IL17a, Csf2, Lta*), IFN related genes (*Ifna2, Ifnb1, Ifnar1*, and *Ifngr1*), and the genes that encode proteins for the inflammasome complex (*Nlrc4, Aim2* and *Casp1*).

Comparing the global results, it can be concluded that R99 but not strain YJ016 induces an early and strong inflammatory response that could be compatible with a CKS.

### RtxA1_3_, but not RtxA1_1_, induces a strong early immune response against *V. vulnificus* in mice

We hypothesized that RtxA1_3_ is a key virulence factor in sepsis. To examine this, we compared the immune response of mice against strain R99 and its derivative mutant R99ΔΔ*rtxA1*_*3*_ at 4 hpi. In parallel, we also compared the immune response of mice against strains YJ016 and YJ016Δ*rtxA1*_*1*_.

Prior to RNA analysis, we checked again the colonization process of internal organs by isolating the inoculated strain from spleen and liver of infected mice. Again, we recovered pure cultures of all the strains, including both mutants, from all the organs and sampling times, but only after enrichment.

In parallel, we checked that *rtxA1*_*3*_ and *rtxA1*_*1*_ were being expressed *in vivo* at 4 hpi, a time at which blood was sampled for transcription analysis. As expected, both bacterial genes were transcribed *in vivo* although with different fold-change values (average fold-change of 2.5 [*rtxA1*_*3*_] vs. 4.8 [*rtxA1*_*1*_]). In addition, we followed the time course of *rtxA1*_*3*_ and *vvhA* transcription to discard the possibility that both toxins could act together *in vivo* at the selected sampling time. *rtxA1*_*3*_ was detected at 2 hpi, being up-regulated at 4 (average fold-change of 2.5) and 6 (average fold-change of 3.0) hpi, while *vvhA* was only induced at 6 hpi although with a higher fold-change (average fold-change 4.0). These results prove that of the two toxins, only *rtxA1*_*3*_ is relevant at 4 hpi.

**Figure 6:**
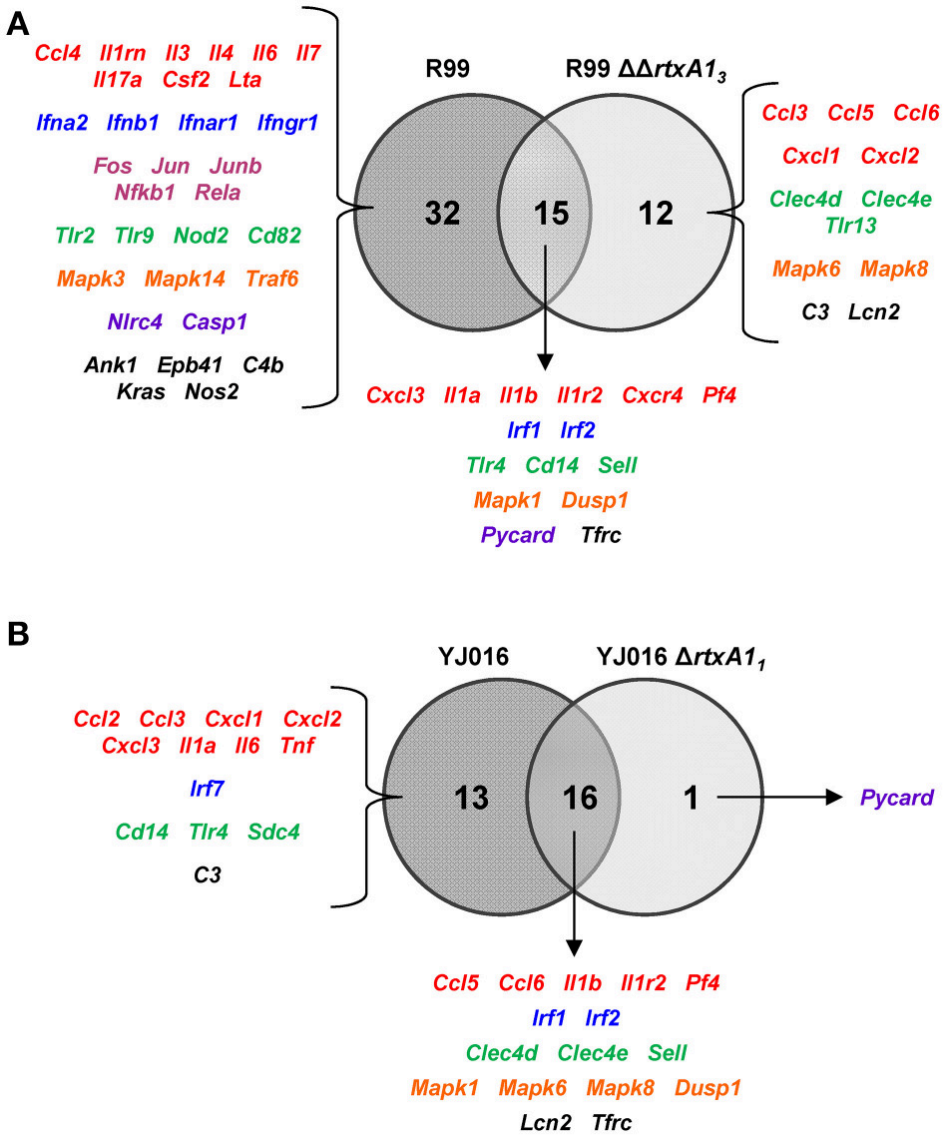
Role of RtxA1 in the induction of the host immune response and CSK. Venn diagram depicting the overlap of the genes that are differently up-regulated between **(A)** R99 and R99ΔΔ*rtxa1*_*3*_, and **(B)** YJ016 and YJ016Δ*rtxA1*_*1*_ infected-mice for 4 h. Only genes up-regulated more than 1.3 were considered.

We then compared the immune response generated by each of the wild-type strains with that induced by its mutant (Figure 6). The R99 strain specifically induced the expression of a large number of genes (32) including those for CKs (*Il3, Il4, Il6, Il7, Il17a, Csf2*, and *Lta*), for IFN-related proteins (*Ifna2, Ifnb1, Ifnar1*, and *Ifngr1*), for inflammasome components (*Nlrc4* and *Casp1*) and for a large number of transcription factors (*Fos, Jun, Junb, Nfkb1*, and *Rela*) (Figure 6A). In contrast, there were significantly fewer differences between the responses generated by the wild-type YJ016 strain and its mutant (Figure 6B); only 13 genes were induced by the wild-type strain and not by the mutant, and the great majority of them (9/13) were also induced by the R99 strain, the R99ΔΔ*rtxA1*_*3*_ mutant, or both (*Ccl3, Cxcl1, Cxcl2, Cxcl3, Il1a, Il6, Cd14, Tlr4*, and *C3*). The strong pro-inflammatory response induced by the R99 strain and not by its mutant would suggest that the activation of the CKS was mainly due to the RtxA1_3_ toxin.

**Figure 7:**
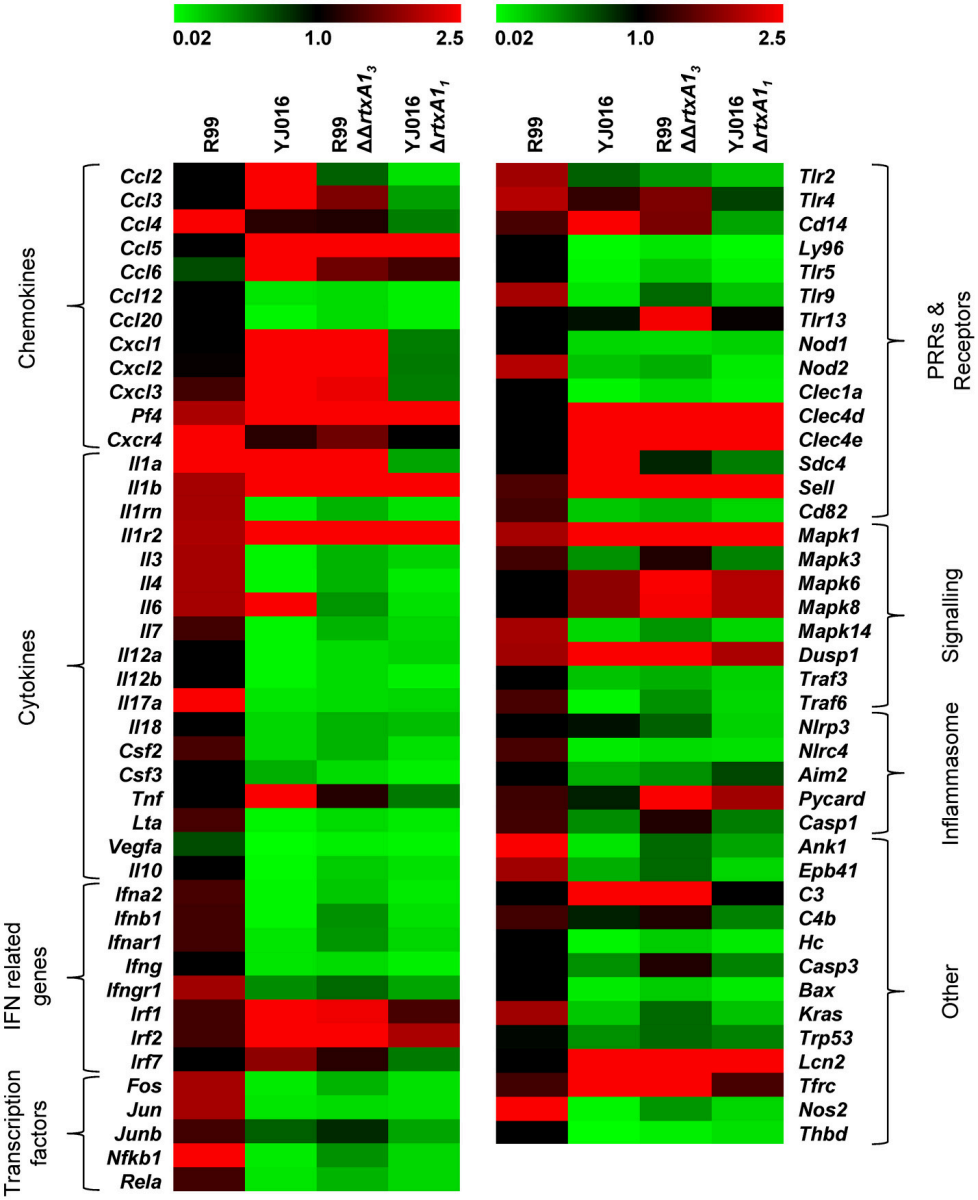
Heat Map. Heat map representing color-coded expression levels of the 84 genes analyzed in infected mice for 4 h with strains Bt2-SerE R99 and Bt1-RtxA1_1_ YJ016, and their derivative RtxA1 mutants. Genes are arranged in functional categories.

We then globally compared the results of the qPCR array from the 4 groups of inoculated animals by performing a Heat Map (Figure 7). The pattern of gene expression induced by both YJ016Δ*rtxA1*_*1*_ and R99ΔΔ*rtxA1*_*3*_ strains was very similar, with a significant number of genes down-regulated (Figure 7). Further, the pattern of gene expression induced by both *rtxA1* mutants was more similar to the one induced by YJ016 strain than to the one induced by strain R99 (Figure 7).

Only 7 out of the 84 selected genes were up-regulated by all four strains; (i) *Il1b* and *Il1r2*; (ii) the genes for the IFN regulatory factors IRF1 and IRF2; (iii) *Dusp1* and *Mapk1*; (iv) *Sell*, encoding L-selectin, a cell surface adhesion molecule required for binding and subsequent rolling of leucocytes on endothelial cells, facilitating their entry into secondary lymphoid tissues and inflammation sites; (v) *Tfrc*, encoding the transferrin receptor; and (v) *PF4*. This latter gene was by far the one most up-regulated, reaching a 433-fold increase (Table 2).

**Figure 8:**
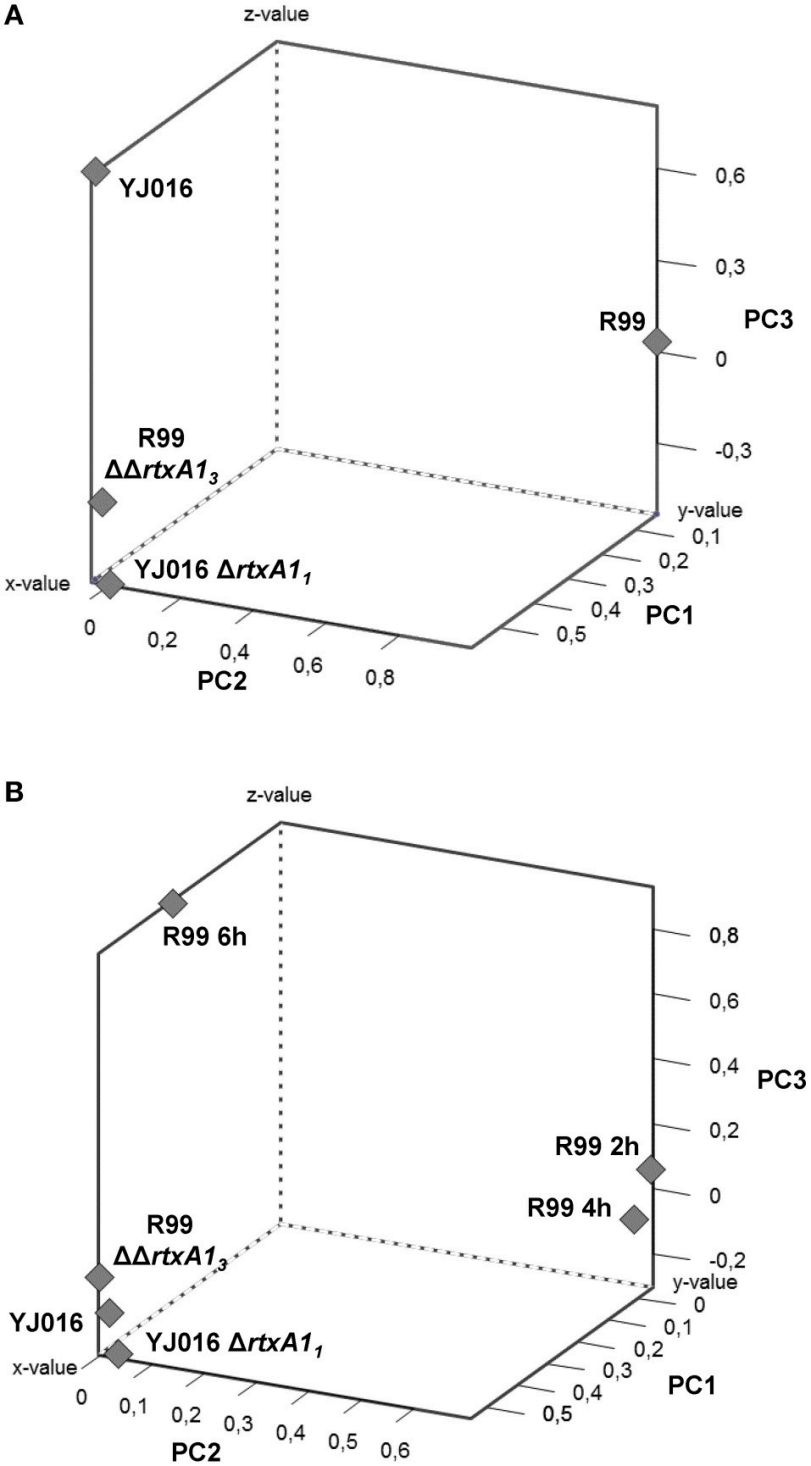
Principal Component Analysis (PCA) 3-D plot of the gene expression. Three principal components are represented, PC1 on X-axis, PC2 on Y-axis, and PC3 on Z-axis. **(A)** shows the analysis of mice infected for 4 h with the wild-type strains and their derivative RtxA1 mutants. In **(B)** mice infected with the Bt2-SerE R99 strain for 2 and 6 h were included in the analysis.

To comprehensively analyse the results, we performed a Principal Component Analysis (PCA) using the data from the mice infected with the wild-type strains and their correspondent mutants, all examined at 4 hpi. As shown in Figure 8A, the analysis confirmed that the immune responses against YJ016 and R99 were clearly different and that the responses against both *rtxA1* mutants were very similar, regardless of the strain that harbored the mutation. Further, the response against R99ΔΔ*rtxA1*_*3*_ was more similar to the one against YJ016 strain than to the one against its own parental strain (Figure 8A), which suggests that the strong immune response against strain R99 was mainly due to RtxA1_3_.

Finally, we repeated the PCA, including the data from the mice infected with strain R99 and analyzed at 2 and 6 hpi (Figure 8B). The analysis grouped immune response data into three clusters; the first corresponding to mice infected with the two *rtxA1* mutants and YJ016 strain, all being analyzed at 4 hpi; the second group corresponded to mice infected with strain R99 and analyzed at 2 and 4 hpi; and the third group corresponded to mice infected with strain R99 and analyzed at 6 hpi. Curiously, this last group was closer to the first one (Figure 8B). This last result, together with the previous ones obtained by PCA, strongly suggests that the immune response due to RtxA1_3_ had a peak at 4 h and decreased at 6 hpi.

### *rtxA1_*1*_* and *rtxA1_*3*_* are expressed in the presence of human blood cells as determine by an *ex vivo* model of hemochromatosis

We used an *ex vivo* model that simulates sepsis in hemochromatosis (hm) human patients and followed the transcription of *rtxA1* genes after the co-culture of the bacteria with human PBMCs (peripheral blood mononuclear cells).

**Figure 9:**
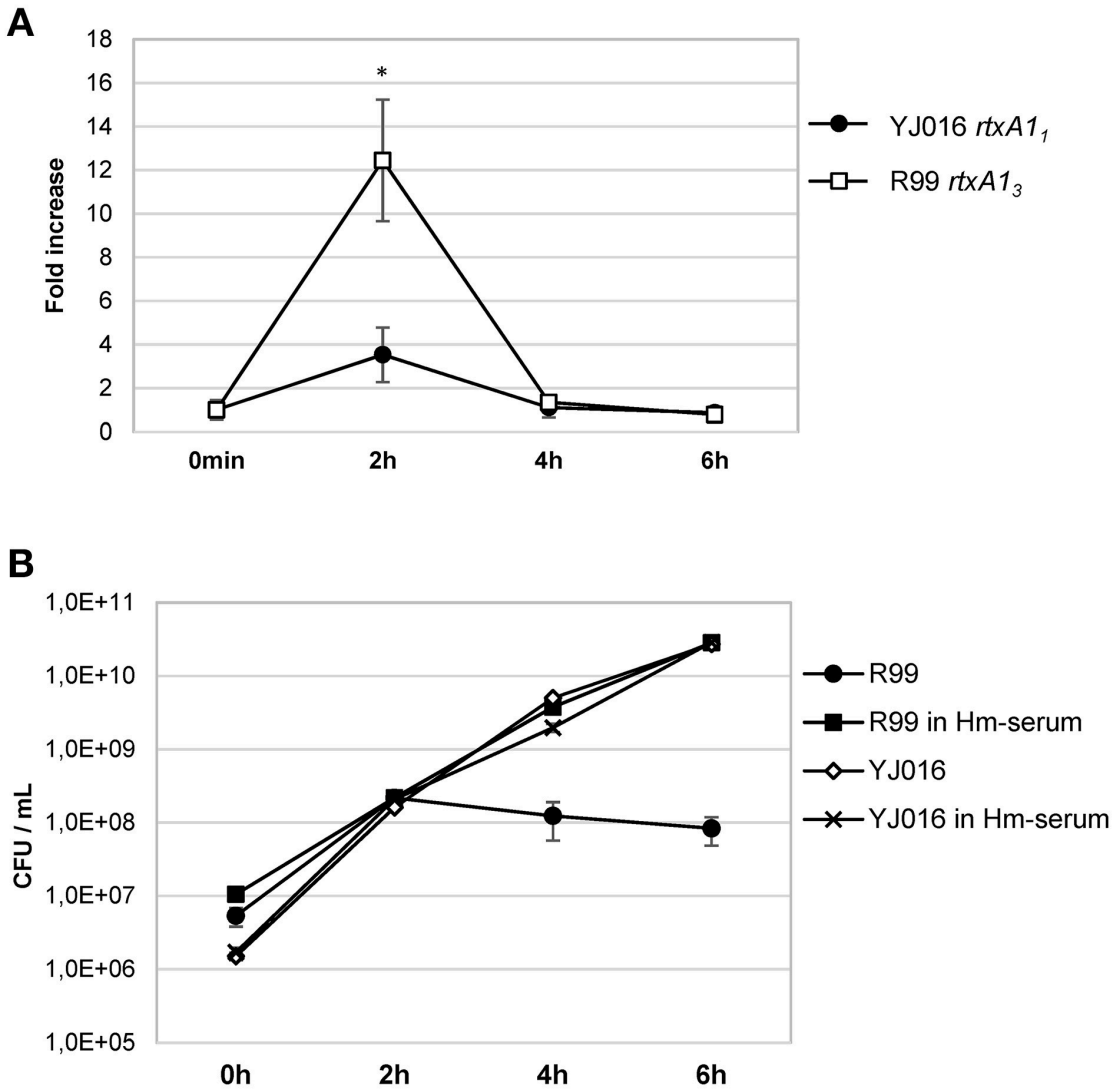
*rtxA1* expression in the presence of human blood cells. **(A)** Expression by Real time qPCR of *rtxA1*_*1*_ and *rtxA1*_*3*_ in YJ016 and R99 strains, respectively. Bacteria were incubated for 0, 2, 4, and 6 h in commercial human serum supplemented with Fe (Hm-serum) plus human PBMCs. The value of expression of each gene in the 0 h sample was set equal to 1, and the fold-change in gene expression was calculated in 2, 4, and 6 h samples. **(B)** Bacterial growth in commercial human serum with (Hm-serum) or without supplemented Fe. **(A, B)** Data represent mean values ± standard deviation from two independent experiments. The significance of the differences was determined using Student's two-tailed *t*-test; ^*^p < 0.05 compared with values at time 0 h.

Real time qPCR results showed that the expression of the *rtxA1* gene was up-regulated in both YJ016 and R99 strains after being in contact with human cells for 2 h. At 4 and 6 hpi, the genes showed similar expression levels as seen at time 0 (Figure 9A). There was a noticeable difference between the expression of *rtxA1*_*1*_ and *rtxA1*_*3*_; *rtxA1*_*3*_ was up-regulated 12-fold when compared with time 0, whereas the *rtxA1*_*1*_ presented a smaller up-regulation (around 3.5-fold).

### *V. vulnificus* Bt2-SerE is unable to overcome blood iron restriction

This last result (Figure 9A) was surprising as YJ016 was selected as a strain representative of the phylogenetic group with the highest virulent potential for humans (Chen et al., 2003). Thus, we checked the growth ability of both strains in human serum, in the presence and absence of a supplemental iron source. Figure 9B shows that strain YJ016 was equally able to multiply in human serum regardless of the presence of supplemental iron. However, strain R99 was able to grow in the first 2 h of incubation, but in the absence of supplemental iron its capacity to multiply disappeared. This finding could explain, at least in part, the higher virulence potential of this phylogenetic group, as strain YJ016 could be able to develop sepsis without predisposing conditions (Chen et al., 2003).

## Discussion

The clinical cases of known-etiology associated with the clonal complex *V. vulnificus* Bt2-SerE correspond to type I human vibriosis after fish handling. The hypothesis underlying the present work refers to the septicemia associated to these vibriosis cases in humans and is based on previous results obtained in the eel which related the RtxA1_3_ with animal death by an early peracute septic shock (Lee et al., 2013). Our hypothesis predicted that once the bacterium passes to blood from an infected wound, and only if the bacterium survives in blood, it would produce RtxA1_3_ that would trigger a CKS resulting in human death. A CKS in the context of a bacterial septicemia could be defined as an unbalanced release of pro-inflammatory and anti-inflammatory CKs in response to the pathogen that results in a severe pathological damage (Tisoncik et al., 2012).

We found that, effectively, the infection of mice with a Bt2-SerE strain caused a strong and early CKS that was directly related to RtxA1_3_ as which was not detected in mice infected with the mutant deficient in the toxin. This CKS was also related to mice death, as mice infected with the mutant strain survived despite being colonized by the mutant. In fact, the wild-type strain spread to the bloodstream and overexpressed *rtxA1*_*3*_ in internal organs as early as 2 hpi, inducing the up-regulation of a significant number of immune system-related genes. This result was also obtained in experiments performed in eels (Lee et al., 2013; Callol et al., 2015a; unpublished results), confirming that Bt2-SerE exhibits very rapid invasion and *rtxA1*_*3*_ *in vivo* expression, whatever the infected host is. In accordance again with results reported in eels (Lee et al., 2013; Callol et al., 2015a), the *rtxA1*_*3*_ deficient mutant was able to reach the internal organs (liver and spleen) so the reduction in immune response intensity was not due to a lack of bacterial stimulus. The peak of immune gene activation against the wild-type strain was reached at 4 hpi, at which time 47 out of the selected 84 genes were up-regulated while none were down-regulated. At this time, the gene encoding the main hemolysin produced by this species, *vvhA*, was not induced. This result confirmed that VvhA was not interfering with RtxA1_3_, in contrast to that reported in orally Bt1-RtxA1_1_-infected mice where RtxA1_1_ and VvhA were found to act together causing pathological damage in the intestine (Jeong and Satchell, 2012).

Interestingly, the CKS induced by RtxA1_3_ included an up-regulation of immune genes related to the response against extracellular and intracellular bacteria. This result was quite unexpected in that all the experiments performed *in vivo* and *in vitro* with strain R99, including those performed in the present work, have never suggested the existence of an intracellular stage in the life cycle of this pathogen in any of its hosts. The unexpected up-regulated genes were for: (i) recognition of intracellular pathogens, such as, TLR9 and NOD2*;* (ii) chemo-attraction of macrophages, natural killer cells, and lymphocytes such as, CCL4 (MIP-1β; Macrophage inflammatory protein-1β), a gene that was highly activated at all sampling points, and that was also early up-regulated in gills from infected eels (Callol et al., 2015a); (iii) regulatory CK such as, LTA (or TNF-β), IL-17 (implicated in triggering and mediating pro-inflammatory response, by inducing the expression of different pro-inflammatory CKs, CCs, antimicrobial peptides, growth factors among others); iv) IFN-related genes (associated with CD8^+^ T cells) such as, *ifna2, Ifnb1, Ifnar1*, and *Ifngr1;* (v) CKs involved in the regulation and stimulation of haematopoiesis such as, IL-3, IL-4, IL-7 and CSF2 (this activation could lead to a rapid innate immune system replenishment, exacerbating the inflammatory response); (vi) the inducible NO synthase gene that mediates tumoricidal and bactericidal actions, mainly intracellular (Maeda and Akaike, 1998); and (vii) members of the inflammasome complex such as, NLRC4, PYCARD, and Caspase-1, that have been previously involved in macrophage death by pyroptosis induced by the facultative intracellular pathogen *Legionella pneumophila* (Case and Roy, 2011; Cerqueira et al., 2015). We also found evidence of a dysregulation of CK production. Thus, DUSP1 (MKP-1) was activated by both strains but was significantly more induced by YJ016 (Table 2). MKP-1 regulates, via dephosphorylation, the activation of several MAPK including p38, thus limiting their signaling potential and the production of pro-inflammatory CKs (Zhao et al., 2006; Wang and Liu, 2007; Moyes et al., 2010). In agreement with the pattern of DUSP1 activation, the *Mapk14* gene (or p38α) was up-regulated by the Bt2-SerE strain, but down-regulated by the selected Bt1-RtxA1_1_ strain. In contrast, mice infected with the Bt1-RtxA1_1_-strain developed a typical immune response against an extracellular pathogen: most of the CKS genes were down-regulated in these mice while genes known to have a beneficial role during extracellular bacterial infections (*Il6, Tnf* or *Lcn2*) were clearly up-regulated (Shin et al., 2002; Berger et al., 2006; Goo et al., 2007; Chuang et al., 2010). Finally, we also found that the Bt1-RtxA1_1_ strain induced high levels of the CCs *Ccl3* and *Cxcl1*. CXCL1 has been recently proven to play a crucial role in the inflammatory response developed against Bt1 *V. vulnificus* infection by reducing hepatic injury (Liu et al., 2015). In contrast, the Bt2-SerE strain only induced these CC at lower levels at 6 hpi, which could contribute to the severity of the disease.

By comparing the immune responses against Bt1-RtxA1_1_ and Bt2-SerE strains we can predict that the common response against any *V. vulnificus* strain would involve, at least, the activation of a series of genes related to LPS recognition (*Tlr4* and *Cd14*) (Kawai and Akira, 2009) together with several genes for the IL-1 family (*Il1a, Il1b, Il1r2*), *IL-6*, and *Pf4* as well as *Tfrc*. Previous works performed with Bt1 strains had already highlighted the important role of TLR4, IL-1α, IL-1β, IL-6, CXCL1, or CCL3 in the response against *V. vulnificus* (Shin et al., 2002; Chuang et al., 2010; Stamm, 2010; Mayer et al., 2014; Liu et al., 2015). However, none of them had reported the activation of either *Tfrc*, a gene encoding a cell surface receptor necessary for transferrin recycling and iron uptake, or *Pf4*. Transferrin is one of the acute-phase proteins that are produced by the liver in response to extracellular bacterial infections, which in turn produce iron-sequestering and a nutritional immunity (Parrow et al., 2013). The activation of *Tfrc* by blood cells would be an indirect signal that transferrin is being produced by the liver. *Pf4*, which was by far the most up-regulated gene by the Bt1 strain, is released by activated platelets, binds to the bacterial surface inducing the formation of neoepitopes, and facilitates bacterial clearance (Krauel et al., 2011). Specifically, PF4 binds to negatively charged LPS on gram-negative bacteria (Krauel et al., 2012). Our results further support the hypothesis that neoepitope formation by PF4 after binding to bacteria is an ancient and early host defense mechanism against extracellular pathogens (Krauel et al., 2012). Nevertheless, both tested *V. vulnificus* strains are encapsulated (Valiente et al., 2008a) and capsule would be expected to interfere with PF4 deposition on LPS, partially explaining why capsulated *V. vulnificus* strains are significantly more virulent than acapsulated ones (Simpson et al., 1987; Strom and Paranjpye, 2000).

Further, we analyzed the immune response pattern by PCA analysis and found that Bt1-RtxA1_1_ and Bt2-SerE MARTX mutants grouped together and close to the Bt1-RtxA1_1_ strain. This proximity in immune response was highlighted even more when the data from R99-infected mice at 2 and 6 hpi were introduced in the analysis; three clusters were observed, one formed by both mutants and the Bt1-RtxA1_1_ strain, another by the samples taken at 2 and 4 hpi from Bt2-SerE–infected mice, and the third with an intermediate position formed from the sample taken at 6 hpi. This finding also suggested that the CKS induced by Bt2-SerE presents a peak very early, at 4 hpi and decreasing at 6 h, a model that is compatible with data of the death dynamic of mice after i.p. infection with Bt2-SerE; most mice either die before 8 hpi or survive the infection.

We had previously demonstrated that *V. vulnificus* Bt2-SerE induced death by lysis of endothelial cells and by apoptosis of human monocytes (Murciano et al., 2015). In our present work, we linked both activities with RtxA1_3_, which suggests that this toxin can induce cell death by different mechanisms depending on the target cell. RtxA1_3_ differs from the other MARTX_Vv_ in that possesses a copy of an ACD domain, in common with MARTX of *V. cholerae*, and two copies of the MCF domain, a domain also present in other MARTX_Vv_ but in only one copy (Lee et al., 2007; Liu et al., 2007; Kim et al., 2008, 2015; Kwak et al., 2011; Lo et al., 2011; Roig et al., 2011; Jeong and Satchell, 2012; Satchell, 2015). Moreover, all the Bt2-SerE strains possess two identical copies of *rtxA1*_*3*_, one in chromosome II and another in the virulence plasmid pVvBt2 (Roig et al., 2011). Thus, we tested if the ACD domain in RtxA1_3_ was also capable of polymerizing actin and found that this domain effectively caused a strong and rapid actin oligomerization *in vitro* in both cell types. However, the ACD domain was not revealed as essential for apoptosis of monocytes *in vitro*, suggesting that the apoptotic activity on monocytes probably was caused by MCF domains present as four copies per bacterium. In partial accordance, it was previously described that MCF domains on other MARTX_Vv_ induce the intrinsic pathway of apoptosis (Agarwal et al., 2015b).

**Figure 10:**
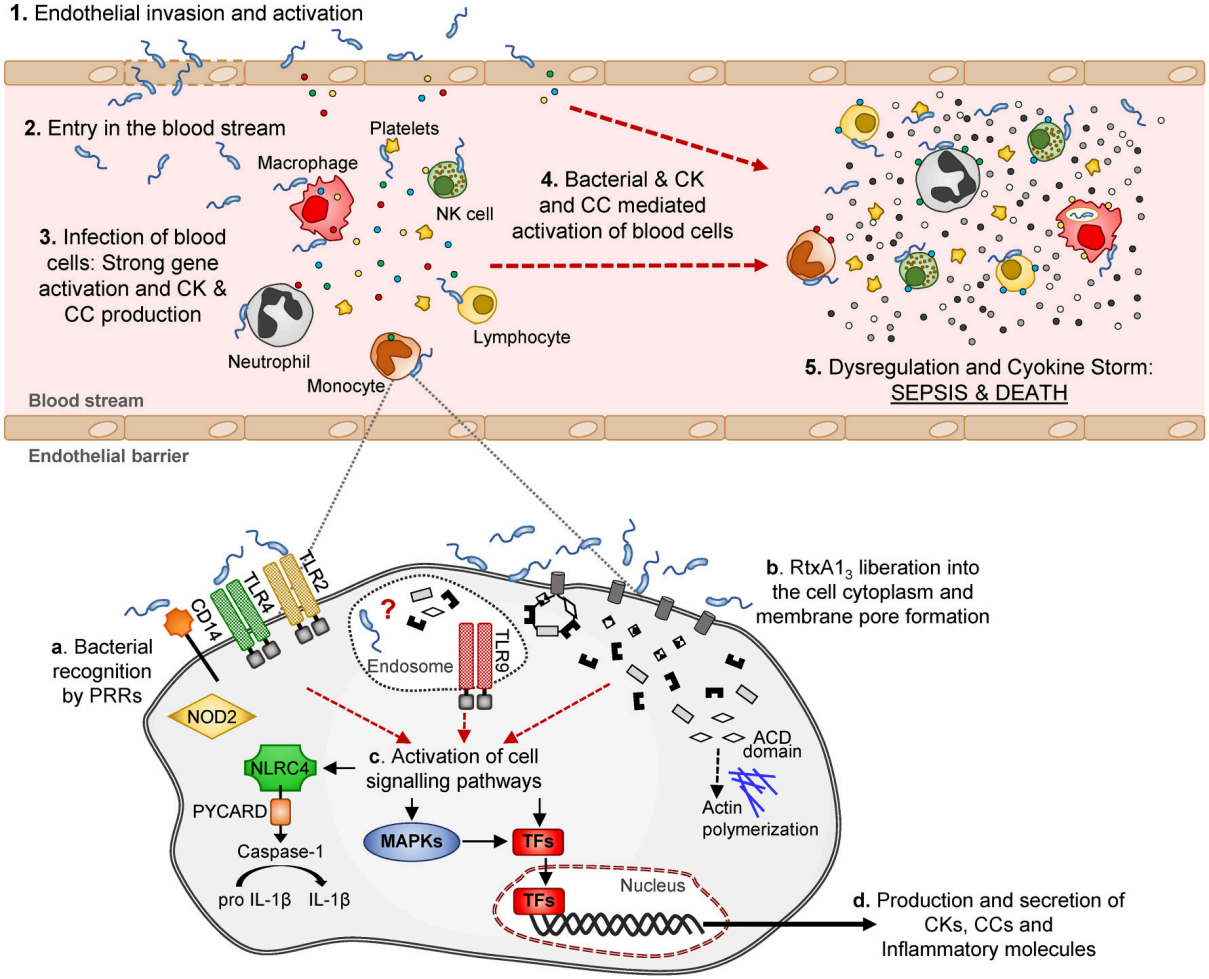
Schematic model of *V. vulnificus* Bt2-SerE induced sepsis. *V. vulnificus* Bt2-SerE would cause sepsis only in patients with high iron levels in blood, and according to the following model: (1) the bacterial cells would reach the endothelial microvasculature from the infection site. There, they will infect the endothelial cells, producing RtxA1_3_ that would enter the host cell and cause death by lysis, allowing the bacterium to invade the blood stream (2). (3) There, the bacterial cells, in low number, would interact with different types of immune blood cells. Once in contact, the bacteria would produce and again liberate RtxA1_3_ into the host cell cytoplasm (see detail in the lower part of the figure). This toxin, together with a superficial recognition of bacterial cells by extracellular and intracellular receptors, would induce activation of different signaling pathways and transcription factors that in turn would produce the activation of apoptosis and/or inflammasome and the secretion of CKs, CCs, and other immune mediators. Although, intracellular receptors are activated, it is not clear if the bacterium is able to enter into the cell cytoplasm or if this activation is triggered by RtxA1_3_ domains or other bacterial components. Thus, the hypothetic intra-cellular life style of *V. vulnificus* Bt2-SerE and/or the mechanism for this anomalous immune response activation remains to be investigated. (4) The inflammatory proteins in response to the infection would recruit and activate additional immune cells. These new cells, which could also be infected with bacteria, would be activated to such a great extent that they will suffer gene dysregulation and a CSK would start, which in turn would produce the host's death by sepsis (5).

Looking in depth the immune-pattern specific for RtxA1_3_ and the mode of action of this toxin, we conclude that, whatever the attacked cell is, either the toxin facilitates bacterial invasion or, alternatively, the toxin by itself activates an immune response that, at least to some extent, is similar to that produced against intracellular pathogens like *L. pneumophila*. Regarding the first possibility, we have never seen bacteria inside eukaryotic cells either in the experiments performed in previous works or in these experiments. We tested the second possibility *in vitro* by infecting the two human cell lines with R99 and its mutant and analyzing the expression of selected CK and CC genes. Regrettably, cells infected with the wild-type strain died early, which resulted in an apparently stronger immune response against the mutant (results not shown), and suggesting that this *in vitro* approach was not adequate to test the hypothesis. In any case and whatever the CKS-activation mechanism is, the fact that this toxin is linked to this storm is exciting, as RtxA1_3_ could be representative of a new family of toxins able to trigger a life threatening immune-response. Figure 10 summarizes a hypothetical model for sepsis induced by RtxA1_3_ in which we propose that not only blood cells but also endothelial cells would be involved. Previously we had demonstrated that endothelial cells can produce, *in vitro*, multiple inflammatory mediators when they are infected with *V. vulnificus* Bt2-SerE (Murciano et al., 2015) This would enhance the destructive effect on mediators liberated by blood cells. Finally, we discarded the possibility that this early CKS was due to bacterial growth in blood; bacteria were present in internal organs of infected mice below the detection limit (<10 CFU/mL) since they could be only recovered after enrichment.

Our next step was to find out if *rtxA1* would be expressed in humans by using an *ex vivo* model of infection. We found that both *rtxA1*_*3*_ and *rtxA1*_*1*_ were highly expressed at 2 hpi in human blood, confirming the results obtained in the mouse. We completed these experiments by growing the bacteria in normal and hm-serum. Interestingly, the Bt2-SerE strain was only able to grow in hm-serum while YJ016 grew in both normal and hm-serum. It is generally assumed that *V. vulnificus* is only able to cause death by sepsis in patients with high iron-levels in blood, although there are a few clinical cases of sepsis in healthy patients described in the literature. YJ016 was selected for the study because it is representative of the phylogenetic lineage that groups the most virulent Bt1 strains, most of them linked to primary septicaemia after raw seafood ingestion (Cohen et al., 2007; Sanjuán et al., 2011). According to our results, YJ016 would also be representative of the group of *V. vulnificus* strains able to cause sepsis in healthy patients.

Epidemiological data reveal that the number of cases of sepsis caused by Bt1 strains is by far much higher than those caused by Bt2-SerE, which never has been linked to primary sepsis after oyster ingestion. These data could lead us to underestimate the public health danger that represents this zoonotic group. In fact, a clinical Bt1 strain has been shown to carry an RtxA1 toxin with the same domain organization as the Bt2-SerE strain, although that strain only had the chromosomal copy of the toxin as it did not have any plasmids (Kwak et al., 2011). This further demonstrates the risk potential of RtxA1_3_ for humans. At the moment, it seems that its geographical distribution is more restricted (Amaro et al., 2015), and its apparent inability to colonize oysters (Amaro et al., 2015) prevents it from being associated with a higher number of clinical cases.

*V. vulnificus* Bt2 shows an extraordinary ability to adapt to the environment, i.e., after substituting saline water by freshwater in eel-farms to control vibriosis, a new Bt2-serovar, able to survive and infect in freshwater, emerged in <5 years (Fouz et al., 2006). Climate change is increasing water temperature and decreasing water salinity, which could extend the geographical distribution of Bt2-SerE and increase the probability of contact with its intermediate reservoir, oysters, and with its end host, humans. This fact together with the fast adaptation of this Bt to external changes suggests a complicated picture for the next years.

In conclusion, for the first time, and by using a murine model of infection, we have demonstrated that the sepsis from vibrosis caused by the Bt2-SerE of *V. vulnificus* could occur through the type III MARTX_Vv_ toxin, which induces an erroneous and dysregulated immune response that would lead to a rapid CKS and, in the end, to death of its host. The specific pattern of the immune response associated to this CKS revealed that the pathogen has either an unknown intra-cellular life stage or, which seems more probable, that the toxin triggers a life-threatening immune response after attacking different immune cells. Although the precise molecular mechanism for the toxic action remains to be determined, we predict that the toxigenic potential of RtxA1_3_ is probably not due to the effect of a single domain (ACD vs. MCF) but to the unique combination of domains (ACD + 2MCF) along with duplication of the gene in the bacterium. This statement is based on previous results obtained with a different mouse strain in which we found that deletion of only one copy of the *rtxA1*_*3*_ gene, either the plasmidic or the chromosomic one, only reduced virulence by 1-log (Lee et al., 2013), and on the results shown here, the expression level of *rtxA1*_*3*_ in human blood being four times the expression level of *rtxA1*_*1*_. Finally, even though the clinical signs of sepsis caused by Bt1-RtxA1_1_ and Bt2-SerE could converge, the mechanisms by which death occurs would be different. Bt2-SerE would induce a dysregulated immune response and a septic shock in which RtxA1_3_ would be critical, while the sepsis caused by Bt1-RtxA1_1_ would be similar to that induced by other gram-negative bacteria, being linked to growth in blood and LPS liberation (Opal, 2010; Ramachandran, 2014).

## Author contributions

CL and TH obtained the mutants and characterized them genetiaclly and phenotypically under the supervision of LH. CM and CA designed the experimental work to test the hypothesis. CM carried out the *in vitro* (assisted by AF), *ex vivo* and *in vivo* (assisted by BF) experiments under the supervision of CA. CM analyzed the results obtained with the appropriate software. CM, CA, and LH discussed the results obtained. CM wrote the draft of the paper, CA corrected the draft and wrote the final version with the final consensus of all the authors.

### Conflict of interest statement

The authors declare that the research was conducted in the absence of any commercial or financial relationships that could be construed as a potential conflict of interest.
