# Supplementary material for: MARTX Toxin in the Zoonotic Serovar of Vibrio vulnificus Triggers an Early Cytokine Storm in Mice
Source: Front Cell Infect Microbiol. 2017 Jul 20;7:332. doi: 10.3389/fcimb.2017.00332 (PMC5517466; doi:10.3389/fcimb.2017.00332)
Supplement: Supplementary file 1 [file Table1.docx]

**Supplementary Table 1. Primers and plasmids used in this study**

| **PRIMERS** | | | |
| --- | --- | --- | --- |
| **Primer** | **Sequence (5’-3’)** | | **Use** |
| recA-forward | CGCCAAAGGCAGAAATCG | | Real-time qPCR. *V. vulnificus* *recA* housekeeping gene |
| recA-reverse | ACGAGCTTGAAGACCCATGTG | |  |
| VvhA-forward | TGTTTATGGTGAGAACGGTGACA | | Real-time qPCR. *V. vulnificus* *vvhA* gene |
| VvhA-reverse | TTCTTTATCTAGGCCCCAAACTTG | |  |
| rtxA1_3_-forward | GAGTGATGATGGGCGCTTTAC | | Real-time qPCR. *V. vulnificus* *rtxA1_3_* gene |
| rtxA1_3_-reverse | CAGCCGCGATGAGATGCT | |  |
| rtxA1_1_-forward | TTAAAGGCAACGCATTTGCA | | Real-time qPCR. *V. vulnificus* *rtxA1_1_* gene |
| rtxA1_1_-reverse | CCGCCCCAACGCATTA | |  |
| B2m-forward | TTCTGGTGCTTGTCTCACTGA | | DNA contamination checking. Murine beta-2 microglobulin |
| B2m-reverse | CAGTATGTTCGGCTTCCCATTC | |  |
| Gusb-forward | GGCTGGTGACCTACTGGATTT | | DNA contamination checking. Murine glucuronidase beta gene |
| Gusb-reverse | GGCACTGGGAACCTGAAGT | |  |
| vvhA-1  vvhA-2 | CCCGACAATAATGGCAGC  GTTCTAGACTTATTTTCCCTCAGATTGG | | Generation of ΔΔ*rtxA1_3_*Δ*vvhA* and Δ*vvhA* mutants (the restriction enzyme site is underlined) |
| vvhA-3  vvhA-4 | GCTCTAGACGTTGGAAACCCACATTA  GGTCTAGAGCCCATTCGTATAGGAAG | | Generation of ΔΔ*rtxA1_3_*Δ*vvhA* and Δ*vvhA* mutants |
| gp018  gp019 | AAAGAGCTCTTCTCATCGTCTGGTGAC  CGGGGTACCTAAGTTCACCCCTGAAAT | | Generation of ΔΔACD mutant |
| gp020  gp021 | CCCGGTACCTCAACACAAACGACGGTA  TGCTCTAGACAACGGGCTAAGAGATTCT | | Generation of ΔΔACD mutant |
| **PLASMIDS** | | | |
| **Designation** | | **Description** | **Isolation source/reference** |
| pGEMT®-easy vector | | Cloning vector, Ap^r^ | Promega |
| pCVD442 | | Cloning vector, *mob* RP4, *sacB*, and Ap^r^ | (Philippe et al., 2004) |
| pUC19 | | Cloning vector, Ap^r^ | (Yanisch-Perron et al., 1985) |
