# Supplementary material for: MARTX Toxin in the Zoonotic Serovar of Vibrio vulnificus Triggers an Early Cytokine Storm in Mice
Source: Front Cell Infect Microbiol. 2017 Jul 20;7:332. doi: 10.3389/fcimb.2017.00332 (PMC5517466; doi:10.3389/fcimb.2017.00332)
Supplement: Supplementary file 2 [file Table2.docx]

**Supplementary Table 2**. **Customized ExProfile^TM^ Gene qPCR Arrays Layout**

| **Plate Position** | **Accession** | **Gene name** | **Gene symbol** | **PCR size** |
| --- | --- | --- | --- | --- |
| **A1** | NM_001013779.2 | Absent in melanoma 2 | *Aim2* | 164 |
| **A2** | NM_001110783.3 | Ankyrin-1 | *Ank1* | 131 |
| **A3** | NM_007527.3 | BCL2-associated X protein | *BAX* | 110 |
| **A4** | NM_009778.2 | Complement component 3 | *C3* | 140 |
| **A5** | NM_009780.2 | Complement component 4B | *C4b* | 109 |
| **A6** | NM_010406.1 | Hemolytic complement | *Hc* | 129 |
| **A7** | NM_009807 | Caspase 1 | *Casp1* | 100 |
| **A8** | NM_009810.3 | Caspase 3 | *Casp3* | 109 |
| **A9** | NM_011333 | Chemokine (C-C motif) ligand 2 | *Ccl2* | 115 |
| **A10** | NM_011337 | Chemokine (C-C motif) ligand 3 | *Ccl3* | 110 |
| **A11** | NM_013652 | Chemokine (C-C motif) ligand 4 | *Ccl4* | 121 |
| **A12** | NM_013653 | Chemokine (C-C motif) ligand 5 | *Ccl5* | 104 |
| **B1** | NM_009139.3 | Chemokine (C-C motif) ligand 6 | *Ccl6* | 95 |
| **B2** | NM_011331.2 | Chemokine (C-C motif) ligand 12 | *Ccl12* | 124 |
| **B3** | NM_016960.2 | Chemokine (C-C motif) ligand 20 | *Ccl20* | 146 |
| **B4** | NM_009841 | CD14 antigen | *Cd14* | 128 |
| **B5** | NM_001136055.2 | CD82 antigen | *Cd82* | 137 |
| **B6** | NM_175526.3 | C-type lectin domain family 1, member a | *Clec1a* | 114 |
| **B7** | NM_019948.2 | C-type lectin domain family 4, member e | *Clec4e* | 104 |
| **B8** | NM_010819.4 | C-type lectin domain family 4, member d | *Clec4d* | 141 |
| **B9** | NM_009969.4 | Colony stimulating factor 2 (granulocyte-macrophage) | *Csf2* | 91 |
| **B10** | NM_009971.1 | Colony stimulating factor 3 (granulocyte) | *Csf3* | 96 |
| **B11** | NM_008176.2 | Chemokine (C-X-C motif) ligand 1 | *Cxcl1* | 149 |
| **B12** | NM_009140.2 | Chemokine (C-X-C motif) ligand 2 | *Cxcl2* | 130 |
| **C1** | NM_203320.3 | Chemokine (C-X-C motif) ligand 3 | *Cxcl3* | 152 |
| **C2** | NM_009911.3 | Chemokine (C-X-C motif) receptor 4 | *Cxcr4* | 145 |
| **C3** | NM_013642.3 | Dual specificity phosphatase 1 | *Dusp1* | 153 |
| **C4** | NM_183428.3 | Erythrocyte membrane protein band 4.1 | *Epb41* | 141 |
| **C5** | NM_010234.2 | FBJ osteosarcoma oncogene | *fos* | 143 |
| **C6** | NM_008337 | Interferon gamma | *Ifng* | 136 |
| **C7** | NM_010503.2 | Interferon alpha 2 | *Ifna2* | 142 |
| **C8** | NM_010508 | Interferon (alpha and beta) receptor 1 | *Ifnar1* | 92 |
| **C9** | NM_010510 | Interferon beta 1, fibroblast | *Ifnb1* | 143 |
| **C10** | NM_010511.2 | Interferon gamma receptor 1 | *Ifngr1* | 116 |
| **C11** | NM_008390 | Interferon regulatory factor 1 | *Irf1* | 158 |
| **C12** | NM_008391.4 | Interferon regulatory factor 2 | *Irf2* | 170 |
| **D1** | NM_016850 | Interferon regulatory factor 7 | *Irf7* | 102 |
| **D2** | NM_010554.4 | Interleukin 1 alpha | *Il1a* | 126 |
| **D3** | NM_008361.3 | Interleukin 1 beta | *Il1b* | 116 |
| **D4** | NM_010555.4 | Interleukin 1 receptor, type II | *Il1r2* | 142 |
| **D5** | NM_031167.4 | Interleukin 1 receptor antagonist | *Il1rn* | 134 |
| **D6** | NM_021283 | Interleukin 4 | *Il4* | 140 |
| **D7** | NM_031168.1 | Interleukin 6 | *Il6* | 141 |
| **D8** | NM_008371.4 | Interleukin 7 | *Il7* | 125 |
| **D9** | NM_010548.1 | Interleukin 10 | *Il10* | 150 |
| **D10** | NM_008351.1 | Interleukin 12A | *Il12a* | 113 |
| **D11** | NM_008352.2 | Interleukin 12B | *Il12b* | 107 |
| **D12** | NM_010556.4 | Interleukin 3 | *Il3* | 95 |
| **E1** | NM_010552.3 | Interleukin 17A | *Il17a* | 118 |
| **E2** | NM_008360.1 | Interleukin 18 | *Il18* | 133 |
| **E3** | NM_010591 | Jun oncogene | *Jun* | 112 |
| **E4** | NM_008416.3 | Jun-B oncogene | *Junb* | 91 |
| **E5** | NM_021284.6 | v-Ki-ras2 Kirsten rat sarcoma viral oncogene homolog | *Kras* | 90 |
| **E6** | NM_008491.1 | Lipocalin 2 | *Lcn2* | 136 |
| **E7** | NM_010735.2 | Lymphotoxin A | *Lta* | 134 |
| **E8** | NM_016923 | Lymphocyte antigen 96 | *Ly96* | 140 |
| **E9** | NM_001038663.1 | Mitogen-activated protein kinase 1 | *Mapk1* | 132 |
| **E10** | NM_011952.2 | Mitogen-activated protein kinase 3 | *Mapk3* | 93 |
| **E11** | NM_015806.5 | Mitogen-activated protein kinase 6 | *Mapk6* | 110 |
| **E12** | NM_016700 | Mitogen-activated protein kinase 8 | *Mapk8* | 125 |
| **F1** | NM_011951.2 | Mitogen-activated protein kinase 14 | *Mapk14* | 145 |
| **F2** | NM_008689 | Nuclear factor of kappa light polypeptide gene enhancer in B-cells 1, p105 | *Nfkb1* | 133 |
| **F3** | NM_001033367.3 | NLR family, CARD domain containing 4 | *Nlrc4* | 127 |
| **F4** | NM_145827.3 | NLR family, pyrin domain containing 3 | *Nlrp3* | 115 |
| **F5** | NM_172729.3 | Nucleotide-binding oligomerization domain containing 1 | *Nod1* | 143 |
| **F6** | NM_145857.2 | Nucleotide-binding oligomerization domain containing 2 | *Nod2* | 111 |
| **F7** | NM_010927.3 | Nitric oxide synthase 2, inducible | *Nos2* | 108 |
| **F8** | NM_019932.4 | Platelet factor 4 | *Pf4* | 143 |
| **F9** | NM_023258 | PYD and CARD domain containing | *Pycard* | 108 |
| **F10** | NM_009045.4 | V-rel reticuloendotheliosis viral oncogene homolog A (avian) | *Rela* | 111 |
| **F11** | NM_011346.2 | Selectin, lymphocyte | *Sell* | 139 |
| **F12** | NM_011521.2 | Syndecan-4 | *Sdc4* | 95 |
| **G1** | NM_009378.3 | Thrombomodulin | *Thbd* | 159 |
| **G2** | NM_011905.3 | Toll-like receptor 2 | *Tlr2* | 172 |
| **G3** | NM_021297 | Toll-like receptor 4 | *Tlr4* | 157 |
| **G4** | NM_016928.2 | Toll-like receptor 5 | *Tlr5* | 102 |
| **G5** | NM_031178.2 | Toll-like receptor 9 | *Tlr9* | 141 |
| **G6** | NM_205820.1 | Toll-like receptor 13 | *Tlr13* | 142 |
| **G7** | NM_011640.3 | Cellular tumor antigen p53 | *Trp53* | 112 |
| **G8** | NM_011638.4 | Transferrin receptor | *Tfrc* | 134 |
| **G9** | NM_013693.2 | Tumor necrosis factor | *Tnf* | 147 |
| **G10** | NM_011632.3 | TNF receptor-associated factor 3 | *Traf3* | 142 |
| **G11** | NM_009424.2 | Tnf receptor-associated factor 6 | *Traf6* | 142 |
| **G12** | NM_009505.4 | Vascular endothelial growth factor A | *Vegfa* | 141 |
| **H1** | NM_007393.1 | Actin, beta | *ACTB* | 104 |
| **H2** | NM_009735 | Beta-2 microglobulin | *B2m* | 112 |
| **H3** | NM_008084.3 | Glyceraldehyde-3-phosphate dehydrogenase | *GAPDH* | 125 |
| **H4** | NM_010368.1 | Glucuronidase, beta | *Gusb* | 148 |
| **H5** | NM_008302.3 | Heat shock protein 90 alpha (cytosolic), class B member 1 | *Hsp90ab1* | 134 |
| **H6** | NM_023281.1 | succinate dehydrogenase complex, subunit A, flavoprotein (FP) | *Sdha* | 133 |
| **H7** | MGDC | Genomic DNA control | MGDC |  |
| **H8** | MGDC | Genomic DNA control | MGDC |  |
| **H9** | RT | Spike-in reverse transcription control | RT |  |
| **H10** | RT | Spike-in reverse transcription control | RT |  |
| **H11** | PCR | Positive PCR control | PCR |  |
| **H12** | PCR | Positive PCR control | PCR |  |
